# MCC: automated mass and charge curation at the genome scale applied to *C. tuberculostearicum*

**DOI:** 10.1128/spectrum.03200-24

**Published:** 2025-12-31

**Authors:** Reihaneh Mostolizadeh, Finn Mier, Andreas Dräger

**Affiliations:** 1 Bioinformatics and Systems Biology, Justus Liebig University Giessen 9175, Giessen, Germany; 2 Biochemistry and Synthetic Metabolism, Max-Planck-Institute for Terrestrial Microbiology 28310, Marburg, Germany; 3 Molecular Design and Pharmaceutical Biophysics, Institute of Pharmaceutical Sciences, Eberhard Karl University of Tübingen 414318, Tübingen, Germany; 4 Data Analytics and Bioinformatics, Institute of Computer Science, Martin Luther University Halle-Wittenberg 9176, Halle (Saale), Germany; 5 Quantitative Biology Center (QBiC), Institute for Bioinformatics and Medical Informatics (IBMI), Eberhard Karl University of Tübingen 9188, Tübingen, Germany; 6 German Center for Infection Research (DZIF), Tübingen, Germany; Cleveland Clinic Lerner Research Institute, Cleveland, Ohio, USA

**Keywords:** *Corynebacterium tuberculostearicum*, genome-scale metabolic model, mass- and charge-balanced, synthetic nasal medium

## Abstract

For many years, antibiotics reliably protected mankind against bacterial infections, including the respiratory tract colonizer *Corynebacterium tuberculostearicum*. However, the spread of antimicrobial resistance necessitates the search for new treatment options, where the microbiota may play a crucial role. One way to investigate the complex nature of bacteria and their interactions with human hosts or microbiota is through genome-scale metabolic models (GEMs). Constructing GEMs is labor-intensive and time-consuming. We introduce the Python package Mass and Charge Curation (MCC), which implements a new automated algorithm to facilitate mass and charge balancing—one of the most time-consuming reconstruction steps. This package manipulates reconstructions by consolidating data from multiple resources and updating the notes field with relevant changes. It also visually compares draft and curated models, ensuring high-quality metabolic reconstructions. Using MCC, we developed a metabolic reconstruction of *C. tuberculostearicum* strain DSM 44922. The model was improved based on standardization policies, resulting in a functional, well-annotated, high-quality product. We also simulated the organism’s growth in synthetic nasal medium 3 (SNM3). The high-quality model *i*CTUB2024RM consistently resembles growth behavior under realistic conditions in an artificial human nasal environment, enhancing the understanding of *C. tuberculostearicum* and its potential impact on health and disease. The curation process of this model led to the development of the MCC package, which facilitates the mass and charge balancing of arbitrary flux balance constraints models in the SBML format.

**IMPORTANCE:**

The rise of antibiotic resistance has made it essential to explore alternative treatments for bacterial infections, particularly those caused by respiratory tract colonizers like *Corynebacterium tuberculostearicum*. Understanding the metabolic behavior of these bacteria and their interactions with the human host or microbiota is crucial. Genome-scale metabolic models (GEMs) are powerful tools for investigating these interactions, but they are time-consuming to build. Our new Python package, Mass and Charge Curation, automates a crucial step in the GEM reconstruction process—mass and charge balancing—making it more efficient and reliable. By applying this tool, we developed a high-quality, functional metabolic model for *C. tuberculostearicum* (*i*CTUB2024RM), which provides deeper insights into the organism’s growth in a simulated human nasal environment. This work offers a foundation for future research into microbial communities and their role in human health.

## INTRODUCTION

The diverse and healthy microbiota in the human body plays a crucial role in maintaining overall health. With the advent of advanced technologies, our ability to study these microbial communities with increasing precision has immensely improved. While much recent research has concentrated on the human gut microbiota, the nasal microbiota also plays an essential role in human health despite receiving less attention. Chronic rhinosinusitis (CRS), a condition characterized by prolonged nasal and paranasal sinus mucosa inflammation (1), exemplifies the importance of understanding nasal microbiota. CRS is a prevalent condition that significantly impairs patients’ quality of life (2). In the United States alone, CRS affected 29.2 million adults, accounting for 14.2% of the population in 2004 (3), and it impacts approximately 5% of the adult population in Western societies (2). In addition, this condition resulted in annual healthcare costs exceeding 5.8 billion (4). It has been widely recognized that microbiota play a role in the pathophysiology of CRS. However, their exact contribution to the development and severity of the disease remains unclear (5). Among the bacteria inhabiting the human nose, corynebacteria species are particularly prevalent (6).

Among various corynebacteria, *Corynebacterium tuberculostearicum* was first described by Brown et al. (7) called leprosy-derived corynebacteria (LDC), and formally characterized by Feurer et al. (8). This corynebacterium is distinct from most other corynebacteria because it produces tuberculostearic acid (TBSA), which Brown et al. identified for the first time in nine strains tested in 1984. However, their species selection focused on the specific epithet tuberculostearicum to reemphasize that this fatty acid occurs in some corynebacterium species (7). Since then, it has also been detected in other members of the genus *Corynebacterium* (9–11). The cells of *C. tuberculostearicum* are nonmotile, nonspore-forming, gram-positive to gram-variable, and non-acid-fast (7, 8). In the sinuses, *C. tuberculostearicum* has been significantly enriched (5, 12, 13) and is suspected to have pathological potential by mediating rhinosinusitis (12).

Furthermore, combining 16S rRNA gene sequencing with improved phenotypic approaches has led to identifying *C. tuberculostearicum* as a causative agent in mastitis (14). *Corynebacterium tuberculostearicum* commonly colonizes various skin environments, including dry and moist regions (15). It plays a role in skin inflammation and may contribute to chronic inflammatory diseases (15). This bacterium is also multiresistant to antibiotics (16), complicating treatment options for infections it causes. Given its adaptability, understanding the environments in which *C. tuberculostearicum* thrives is essential as it may play a role in maintaining microbial balance and contributing to the host’s immune defense mechanisms (15). Although usually commensal, *C. tuberculostearicum* can act as an opportunistic pathogen, particularly in immunocompromised individuals (17, 18). It has been implicated in various infections, including pneumonia, septic arthritis, and infections associated with medical devices (17, 18). Understanding its metabolic capabilities is crucial for identifying factors that enable its transition from a harmless commensal to a pathogenic state. Moreover, metabolic reconstruction can help identify potential targets for novel antibiotics or alternative therapeutic approaches. This necessitates a focus on a genome-scale metabolic model (GEM), which can efficiently identify and characterize the metabolic systems of nasal microbiota. A GEM is constructed based on genome sequence annotation and physiological data, encompassing all the metabolic reactions within an organism and the genes encoding each enzyme.

A recent study using GEMs on *Corynebacterium* species found that *Corynebacterium glutamicum* serves as an important platform for industrial biotechnology and environmental remediation (19). As noted, Gu et al. describe high-quality, experimentally validated models (e.g., for *Escherichia coli* (20) and *Bacillus subtilis* (20) and *Mycobacterium tuberculosis*) (20) that serve as benchmarks in the field. They also confirmed that the models for *C. tuberculostearicum* are listed in the Assembly of Gut Organisms through Reconstruction and Analysis (AGORA) (21). Furthermore, BioCyc (22) includes a model for *C. tuberculostearicum*. However, all these models were constructed for strain SK 141. The BioCyc model, created in June 2020, provided more recent and detailed annotations than AGORA (February 2019) (21). Therefore, we transferred only those reactions from this BioCyc model with more than 95% sequence identity to extend our model.

Furthermore, a model for the *C. tuberculostearicum* strain FDAARGOS_1117 was identified in the KEGG database (23), created in 2021, using the National Center for Biotechnology Information (NCBI) Reference Sequence (RefSeq) (24) assembly GCF_016728365.1 using culture collection strain DSM 44922. Therefore, this strain might be labeled differently. To analyze the production of TBSA in our model, we integrated relevant information from the BioCyc (22), AGORA (21), and KEGG (23) models. This article describes the first study presenting a high-quality *C. tuberculostearicum* strain DSM 44922 GEM. GEMs play a crucial role in understanding cellular metabolism and phenotypes, designing mutant strains for desired products, and assessing the effects of genetic interventions and environmental changes on cellular metabolism (25). High-quality GEMs provide deeper insights into the species and its interactions with other species, offering more personalized treatment options for diseases caused by them.

High-quality reconstructions of GEMs require extensive manual curation, which is often time-consuming. To streamline this process and reduce the need for manual work, we developed a Python module called Mass and Charge Curation (MCC). This tool automatically curates mass and charge assignments for metabolites in a metabolic model. The algorithm behind the Python module is explained in detail to ensure transparency. The module gathers mass and charge information from various databases, including Biochemical, Genetical, and Genomical (BiGG) (26), MetaNetX (27), KEGG (28), BioCyc (22), and Chemical Entities of Biological Interest (ChEBI) (29), and evaluates the results. If any changes are made to the chemical formula or the number of protons in a reaction to balance the mass, these changes are documented in a notes field within the model.

Additionally, the module can visually compare the draft and curated models. This allows users to see all metabolites with incomplete information or where the assignment differs from the draft model. The MCC package is freely available through a GitHub repository (see Data Availability).

The development of this package was driven by the goal to curate the genome-scale metabolic network reconstructions (GENRE) of *C. tuberculostearicum* strain DSM 44922. To meet the latest community standards, this reconstruction process followed best-practice recommendations by Carey et al. and adhered to the findable, accessible, interoperable, and reusable (FAIR) data principles (30). As a result, the GEM includes a variety of features, such as fully annotated metabolites, reactions, and genes with GPRs, SBO terms (31), Evidence and Conclusion Ontology (ECO) terms (32), Systems Biology Markup Language (SBML) (33) extension package for groups (34), and KEGG (28) pathway annotation. In addition, refinement steps were implemented to address energy-generating cycles (EGCs), redundancy, dead-end metabolites, and model extensions using other resources. These enhancements help fill knowledge gaps regarding growth possibilities in different environments, like Luria-Bertani (LB), M9 minimal medium (M9), and SNM3 (35). The new high-quality GEM for *C. tuberculostearicum* strain DSM 44922 is available in BioModels, providing a comprehensive model description.

## MATERIALS AND METHODS

It is essential to use the latest *in silico* methods and tools to achieve a high-quality GEM. Following the most current standards in systems biology, based on the reconstruction protocol from 2010 by Thiele et al., ensures the model meets these standards.

### Reconstruction

The model reconstruction process was broken down into three main steps: (i) creating a draft model using an open-source and user-friendly tool; (ii) manual curation for improving the model; (iii) the analysis and quality control of the model.

#### Draft model creation

The model was created using a top-down reconstruction approach with CarveMe (36). We build on a universal model since our organism seems gram-variable. CarveMe uses information from the universal model to *carve away* any reactions unlikely to occur in the organism’s proteome.

#### Model refinement

The model created by CarveMe was based on a curated universal model using the BiGG Models Database (26), which includes data from several high-quality, manually curated GEMs. To manipulate chemical formulae and charges of metabolites, we used the SBML Level 3 (37) fbc extension package (38). Manual curation was performed using Constraints-Based Reconstruction and Analysis for Python (COBRApy) (39) and the libSBML (40) API for SBML (33) in Python to improve and fill in any missing information.

##### Database annotation for reactions, metabolites, and genes

Several database annotations, including KEGG (28), BioCyc (22), ChEBI (29), ModelSEED (41), and MetaNetX (27), were extracted from the notes fields using libSBML (40). This process standardized the data to make the model more comparable across different databases, providing a consistent way to track provenance and enabling reuse through uniform formats. This improved the reproducibility and comparability of GEMs across various databases. All annotations were added as controlled vocabulary (CV) terms with the biological qualifier type BQB_IS, using https://identifiers.org through the Minimal Information Required In the Annotation of Models (MIRIAM) registry (42). This approach ensures the annotations remain accurate even if databases are modified (43). We parsed old and new locus tags from the GeneBank file corresponding to our specific strain for gene annotation. Depending on the availability, these tags were organized into a dictionary to map gene identifiers or into a table with WP Numbers and old and new locus tags. We checked which gene identifiers (IDs) were available in our model (probably “WP_...”). Using this information, we annotated the model with COBRApy. To do so, we first gathered the UniProt accession IDs from the old locus tags through https://www.uniprot.org/uploadlists/. We then queried UniProt for additional IDs. With the old locus tags, we also accessed the KEGG database via the KEGG API to retrieve references to further databases. One could use conversion methods to revert to the old tags if only new locus tags were available. Finally, all gene annotations were added to the model using libSBML (40).

##### Extend the model manually using the BioCyc database

The BioCyc database (22) version 26.1 includes metabolic pathways for *C. tuberculostearicum* strain SK141 with Reference Sequence (RefSeq) (24) assembly accession, GCF_000175635.1, and Taxon ID id=553206. This database is continuously updated with new biochemical information, such as from MetaCyc (44), using both computational and experimental approaches. Additionally, the organized assembly of organism information in the NCBI (45), with Reference Sequence (RefSeq) (24) assembly accession GCF_013408445.1, is efficiently extended. As a result, a reconstructed GEM will eventually require updates. Despite the different strains available in BioCyc (22) and our reconstructed model, analyzing these different strains can still yield additional predictive reactions. The extension process was performed as follows:

1. Create a SmartTable with the relevant information using BioCyc (22) and download it.
2. Read the tables and organize the data into dictionaries.
3. Restore information, including reaction details, Enzyme Commission (EC) numbers, reaction direction, reaction names, substrate, and substrate formulae, and map their BioCyc ID to the respective values. Since BioCyc SmartTables do not contain substrate charges, we initially set each charge to 0 in this stage.
4. Parse the protein sequences to blast them against the sequence of our strain.
5. Align the sequences with our strain using DIAMOND (46).
6. Evaluate the alignment results, comparing them to those in our model based on a 95% identity threshold, and restore the results in a dictionary.
7. Extract BioCyc genes for the reactions and compare them to those in our model.
8. Add reactions from BioCyc (22) for which gene evidence was found but were not present in our model.

These steps create an extendable framework applicable to any strain-specific model, making it feasible to combine comparative genomics and genome-scale metabolic modeling across multiple species with reasonable effort and expertise.

##### Mass and charge balance

We extracted all chemical formulae from the notes field into dedicated attributes or MIRIAM annotations within the SBML (33) model with fbc extension (38), created by CarveMe (36), and assigned each species a zero charge. While this results in a perfectly balanced model, it does not accurately reflect the biological reality. Assigning the correct charge can cause issues with imbalanced reactions, and the presence of multiple formulas for some metabolites may also lead to mass imbalances. To improve the model, each metabolite should have a unique chemical formula and correct charge, even though this can increase the number of mass imbalance reactions. A reaction is elementally and charge-balanced once the protonation states of its reactants and products are correctly determined, which may require recovering some charge information. This balancing process can lead to an infinite loop, making it one of the most time-consuming steps during manual curation. To simplify this process and resolve charge assignment and reaction balancing, we developed an automatic Python module called MCC. This module synchronizes refining chemical formulas and associated charges, ensuring balanced reactions while saving time. The details of this approach will be explained in the next section.

##### SBO and ECO terms

To introduce additional semantic information to the model, SBO terms were assigned to all genes, metabolites, and reactions using libSBML (40) in Python. The SBO provides controlled vocabularies commonly used in systems biology (31). By using SBOannotator (47), we assigned the following: (i) the SBO term SBO:0000243 to each geneProduct element, representing the concept *gene;* (ii) the SBO term SBO:0000247 to each metabolite, representing the concept *simple chemical*; (iii) specific SBO terms based on their type. The latter includes, for instance, *exchange*, *sink*, *demand reaction*, *growth*/*biomass reaction*, *transport* (specifically sym- and antiport reactions), *translocation* reactions, simple reactions containing the reactant and product compartments, and specifically *efflux* and *influx* reactions.

Additionally, ECO terms (32) (classes) were included to describe the types of evidence used, from laboratory experiments, computational methods, literature curation, or other means. These terms help track annotation provenance, establish quality control, and provide insights into the sense of “why we believe what we think we know” (32).

##### Groups extension

We enhanced our model by grouping pathways identified via KEGG (28). Using the SBML (33) groups package through libSBML (40) in Python (34), we extracted all reactions annotated with BQB_OCCURS_IN along with their associated pathways. For each pathway, we created a group of type partonomy and added the corresponding reactions, indicating that these reactions are part of a common pathway.

Additionally, we included BQB_IS annotations and CV terms of biological qualifier type BQB_OCCURS_IN to indicate the pathways to which reactions belong. Where necessary, we used web requests in Python to extract missing KEGG (28) reaction identifiers from the BiGG API and then retrieved the associated pathways from KEGG (28) via its REST API. These pathways were then added to each reaction with an annotated KEGG identifier.

##### Energy-generating cycles

Energy metabolism is crucial to cellular biology. Insufficiently curated GEMs can lead to thermodynamically infeasible EGCs or futile cycles without nutrient consumption. Any EGC violates the law of energy conservation, compromising the reliability of model simulations. Therefore, it is essential to accurately account for the use of external nutrients to synthesize energy metabolites in species-specific GEMs. To identify if the model contains any EGCs, we used FBA with zero nutrient uptake while maximizing energy dissipation reactions. This included reactions for adenosine triphosphate (ATP), cytidine triphosphate (CTP), guanosine triphosphate (GTP), uridine triphosphate (UTP), inosine triphosphate (ITP), reduced nicotinamide adenine dinucleotide (NADH), NADPH flavin adenine mononucleotide and dinucleotide, ubiquinol-8, menaquinol-8, 2-demethylmenaquinol 8, acetyl-CoA, L-glutamate, and proton exchange between cytosol and periplasm, as demonstrated by Fritzemeier et al. (48) Such cycles may occur if the GEM lacks constraints on reaction irreversibility or contains erroneous reactions or cofactors. Identified EGCs were eliminated by either constraining the reaction directionality or removing the entire reaction.

### Model analysis

The model was analyzed to identify the factors and small molecules that influence the organism’s growth rate under different environmental conditions. This was achieved by maximizing the biomass composition reaction predicting the growth rate.

#### Growth on varying media

To determine the biomass components needed to sustain growth (49, 50), FBA simulates microbial metabolism under specific environmental conditions (51), such as LB, M9, SNM3, and Brain Heart Infusion (BHI) broth or agar (ATCC Medium 44) supplemented with 1% Tween‑80. LB is a nutritionally rich medium primarily used for bacterial growth (52). M9 is a minimal salt base formulation, often supplemented with amino acids and carbon sources, and is commonly used to cultivate *Escherichia coli* (53). SNM3 is a specialized medium designed for *in vitro* testing systems related to the human nose and supports the growth of bacteria, especially those from the Firmicutes phylum (35). BHI broth is a rich, nutrient-dense medium commonly used to cultivate particular organisms, including many bacteria (54, 55). When supplemented with 1% Tween‑80, it becomes beneficial for organisms that require or benefit from additional lipid components for enhanced growth, such as *Mycobacterium* and *Corynebacterium*. Tween-80 primarily contributes to oleic acid, an essential fatty acid for many bacterial species (56). FBA can also predict nutrient utilization, product secretion, pathway usage, and missing reactions in GEM networks (57).

Furthermore, FBA computes the minimal medium requirements, i.e., the minimum number of essential metabolic reactions needed to support specific growth rates. This minimal medium assesses growth on different carbon sources (58). Each time, the carbon source is swapped out with another available option in the model, and growth is then recalculated.

#### Model quality

Evaluating the efficiency of GEM generation, refinement, and manual curation is challenging. To assess the quality of a GEM and its ability to generate meaningful predictions, the protocol by Thiele et al. suggests using confidence scores to refine a draft model into a high-quality one. ECO terms (32) now replace these scores. While advanced platforms can ease manual modifications, the curation process relies heavily on manual efforts. More time spent on manual curation typically results in a higher-quality model (59). To evaluate the overall quality of a reconstructed GEM, the open-source platform MeMoTe is used for quality control and assurance (60). MeMoTe assigns a score ranging from 0 % to 100%, based on a series of tests on stoichiometric GEMs (60). This score helps determine the model’s quality and transparency improvements.

### Software requirements

The MCC algorithm was implemented in Python (version > 3.8). We also use the Z3 sat-solver (https://github.com/Z3Prover/z3) to identify minimal unsat cores. The ReadMe file within the MCC project’s repository contains all requirements, installation, and usage instructions (see Data Availability).

## RESULTS

The results here are divided into two parts: (i) one focusing on the algorithm behind the Python module MCC for automated MCC and (ii) the other focusing on the reconstruction steps for model *i*CTUB2024RM strain DSM 44922.

### The mass and charge curation algorithm

The chemical formula for the metabolites originated from the notes field of the draft model created by CarveMe, which was pulled from the BiGG Models Database (26). However, BiGG contains some metabolites with multiple chemical formulas, missing information, or undefined side-groups (e.g., alkyl groups -R). Similar issues were found with the charges associated with these metabolites during structural reconciliation. These inaccuracies strongly affected the mass balance of reactions, leading to imbalances or inconsistencies when metabolites with undefined side groups were present. To address these issues, we searched across databases to find the best possible match for each metabolite’s chemical formula and charge. We then paired the affected reactions in the model with database reactions if at least one participating species was involved. Since every metabolite should have an apparent molecular formula, except for the number of hydrogen atoms, which may vary, we separated the nonhydrogen balancing from the hydrogen and charge balancing. The resulting algorithm follows these six steps:

#### Data collection

Determine and sanitize the chemical formulas and charges found in databases. This process can be easily adjusted and extended by writing and registering custom database interfaces.

#### Encoding-based satisfiability modulo theories (SMT)

SMT problems involve determining whether a given logical formula can be satisfied, meaning there is some assignment of values to its variables that makes the formula true (61). These formulas can include Boolean logic, similar to Boolean satisfiability problems (SATs), which focus on finding an interpretation that satisfies a given Boolean formula (62). They can also be more complex mathematical structures or theories.

The SMT encoding adds constraints for each possible metabolite formula and charge as well as every reaction that is part of the model.

##### Chemical formula and charge encoding

To represent possible chemical formulas and charges identified during data collection, we introduce for each metabolite *m*:

$s_{z}^{m}$: integer variable for the assigned charge;
$s_{X}^{m}$: integer variable for the assigned stoichiometric coefficient for element X in m.

Given a set of candidate formulae and charge assignments for metabolite *m*:


$$\{(a_{m}^{i}(z),a_{m}^{i}(X)|X\in E){\}}_{i}$$

We define a Boolean variable $\alpha_{m}^{i}\in A$ that encodes the *i*-th candidate assignment for metabolite m as follows:


$$\alpha_{m}^{i}\equiv \left\{ \begin{array}{ll} (s_{z}^{m}=a_{m}^{i}(z))\wedge \underset{X\in E}{⋀}s_{X}^{m}=a_{m}^{i}(X), & \text{without rest group}\\ (s_{z}^{m}=a_{m}^{i}(z))\wedge \underset{X\in E}{⋀}s_{X}^{m}\geq a_{m}^{i}(X), & \text{with rest group,} \end{array}\right.$$

and omit the charge condition if the charge is unknown. Inequalities ensure that known partial formulas are respected.

For each metabolite m, we define the constraint $P_{m}$. If no assignment can be found, only positive numbers of atoms are allowed:


$$P_{m}\equiv \left\{ \begin{array}{ll} \underset{i}{⋁}\alpha_{m}^{i}, & \text{any assignment found}\\ \underset{X\in E}{⋀}s_{X}^{m}\geq 0, & \text{no assignment found} \end{array}\right.$$

This enforces that either one of the candidate assignments is chosen, or if none are available, only non-negative atom counts are enforced.

##### Reaction encoding

To decouple mass from charge/proton balance, reactions $r=\sum_{i}c_{i}m_{i}$ ⇌ $\sum_{j}c_{j}m_{j}$ are encoded via the following:

- **Mass balance** (excluding protons)
$$M_{r}\equiv \underset{X\in E\setminus \{H\}}{⋀}\sum_{i}c_{i}s_{X}^{m_{i}}=\sum_{j}c_{j}s_{X}^{m_{j}}$$
- **Charge balance** (via proton difference)
$$Z_{r}\equiv \sum_{i}c_{i}s_{H}^{m_{i}}-\sum_{j}c_{j}s_{H}^{m_{j}}=\sum_{i}c_{i}s_{z}^{m_{i}}-\sum_{j}c_{j}s_{z}^{m_{j}}$$

$B_{r}$ is defined as follows to indicate reaction r is balanced:


$$B_{r}\Rightarrow (M_{r}\wedge Z_{r})$$

##### SMT formula

The complete constraints are given as follows:


$$P\equiv \underset{m}{⋀}P_{m}\wedge \underset{r}{⋀}B_{r}$$

The balancing problem is formulated as determining a characteristic function of possible assignments α:A→{0,1}, such that $[\;[P]\;{]}^{\alpha }$ is true.

### Determine the balanceable (SAT) core

Each reaction that appears in an unsat core is removed from the balancing process until a fully balanced subset of reactions is identified. Removed reactions are still considered for proton and charge balancing and retained in the model. Any such reaction that is not reintroduced is marked as unbalanceable.

### Reintroduce unsatisfiable reactions

After identifying the balanceable core, reactions that could not be balanced together were removed. However, some removed reactions may be correct, e.g., those linking incorrect reactions to the system. To address this, each set of mutually unbalanced reactions (unsat cores) is reconsidered by reintroducing reactions individually. A reaction is added back only if it balances with the current set, ensuring consistency with most core reactions. This assumes that most balanceable reactions and their metabolite formulae are accurate, and this accuracy extends transitively to newly added reactions. This greedy heuristic depends heavily on the order of reaction consideration, with prioritization as follows:

- Prioritize small sets of mutually unbalanceable reactions to reduce state explosion.
- Prioritize reactions in the vicinity of manually fixed metabolite formulae.
- Heuristically prioritize reactions based on the number of balancing options for the current set of balanceable reactions.

Thus, any manually added formulae are heavily prioritized.

**Algorithm 1** Reintroduction of reactions

**Definitions:**

- R: a collection of sets of mutually unbalanceable reactions.
- For each *R* ∈ R:
  – G(*R*): a partition of *R* into groups of reactions that share the same distance to any manually fixed metabolite formulae.
  – *σ* : *R* → ℝ: a function that assigns a score to each reaction.
- SAT_Core: Current set of mutually balanceable reactions.

1: **for** each *R* ∈ R (in increasing |*R*|) **do**

2:    **for** each *G* ∈ G(*R*) (in increasing distance) **do**

3:        **for** each *r* ∈ *G* (in decreasing *σ* (*r*)) **do**

4:            **if** SAT(SAT_Core ∪ {*r*}) **then**

5: SAT_Core ← SAT_Core ∪ {*r*}

6: **end if**

7:         **end for**

8:    **end for**

9: **end for**

The scoring heuristic prioritizes reactions that are balanced by formula and charge assignments that are scored to be more likely by the rest of the set of reactions. An assignment is considered more likely if it balances more reactions. The scoring leverages the assumption that only a few reactions and metabolites are genuinely faulty, enabling the rest to identify likely correct candidates. Scoring is done as follows:

Given assignments α:A→{0,1}*,* which assign formulae and charges to all metabolites, we determine a metabolite’s *i*-th potential formula (and charge) assignments $a_{m}^{i}\in A$ scores $\sigma (\alpha_{m}^{i})$ as the sum of balancing assignments *α* per reaction *r* in which the formula assignment is used $\hat{=}\;\alpha (\alpha_{m}^{i})=1$:


$$\sigma^{\prime }(\alpha_{m}^{i})=\sum_{r}\sum_{\alpha \in \{\alpha |[\;[B(r)]\;{]}^{\alpha }\}}\alpha (\alpha_{m}^{i})$$

With


$$[\;[B(r)]\;{]}^{\alpha }\;\hat{=}\;\text{Reaction }r\text{ is balanced given assignment }\alpha$$

Each score is normalized with respect to its corresponding metabolite:


$$\sigma (\alpha_{m}^{i})=\frac{\sigma^{\prime }(\alpha_{m}^{i})}{\sum_{j}\alpha_{m}^{j}}$$

To greedily determine the best reaction to reintroduce, each reaction is assigned a score σ(r) based on its best balancing assignment. The assignment score is determined as the mean square of each of the formula assignment scores to punish particularly unlikely formula assignments.


$$\sigma (r)=\max_{\alpha \;|\;[\;[B(r)]\;{]}^{\alpha }}\frac{\sum_{m\in M(r)}\sigma (\alpha_{m}^{i}{)}^{2}\cdot \alpha (\alpha_{m}^{i})}{|M(r)|}$$

With


$$M(r)\;\hat{=}\;\text{the set of reactants of reaction }r\;\text{.}$$

### Optimization of the assignment

Once the most extensive set of balanced reactions is identified, the model is optimized in three steps:

- Adherence to the original models’ formulae if reactions remain balanced.
- Use the most detailed formula that still keeps the model balanced available.
- If the formula is not constrained, use an unconstrained representation from a database. Otherwise, use a minimal count of atoms.

### Post-processing

- Handle unconstrained formulae. Metabolites with unconstrained formulae (i.e., containing a rest symbol) fall into two cases: (i) the formula can be inferred from the context and is marked accordingly or (ii) the formula remains unknown or represents multiple species, typically appearing alongside another metabolite with a rest symbol (e.g., oxidized/reduced thioredoxin), in which case the rest symbol is retained and the formula is marked as unconstrained.
- When possible, choose hydrogen/charge representations adhering to the original model. If not, opt for the most neutral representation, which seems sensible, similar to KEGG (28).
- Add protons to ensure full mass balance in the reactions.

**Fig 1:**
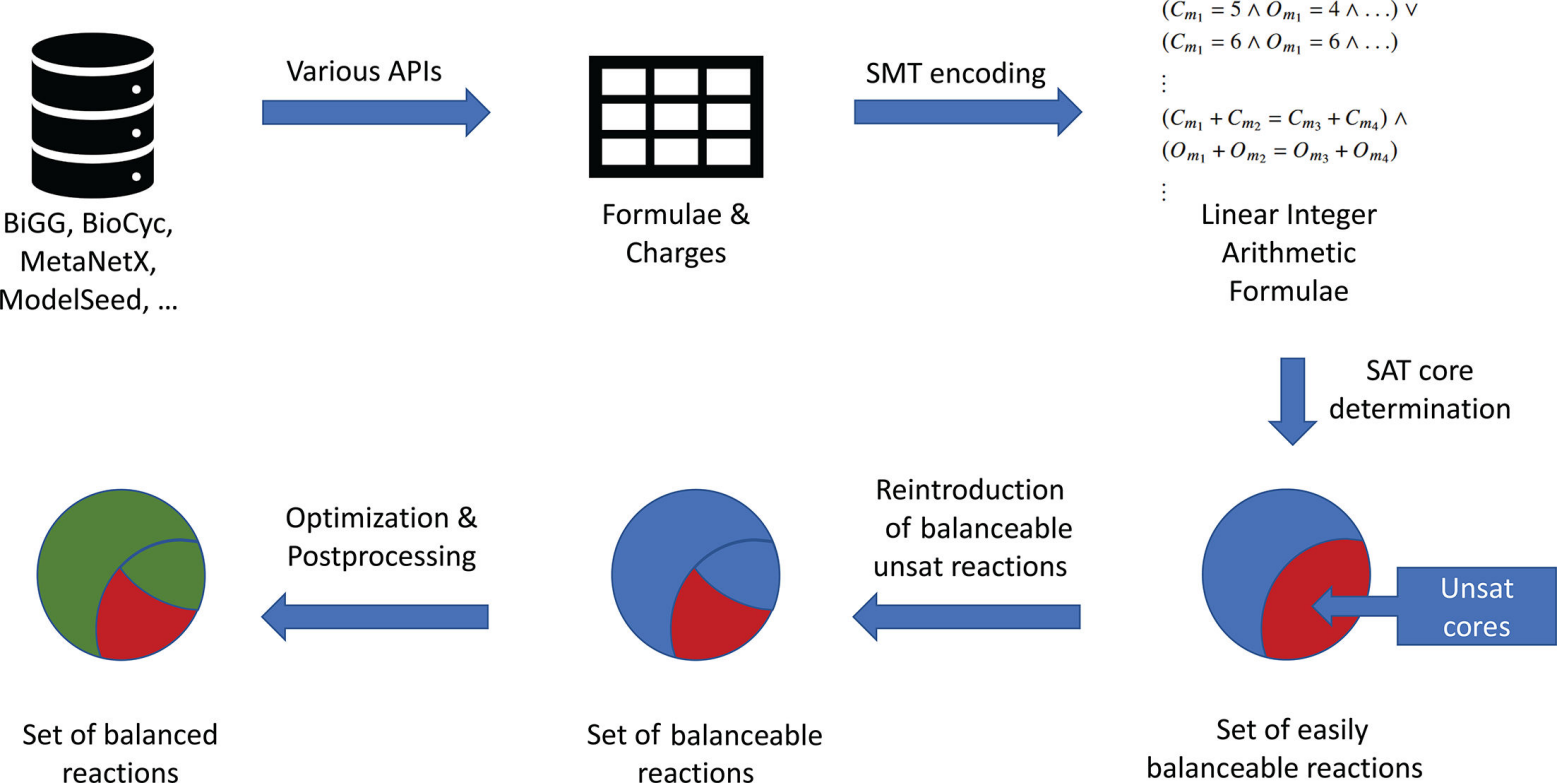
The flowchart of the algorithm used to create the Python module mass and charge curation (MCC).

**Fig 2:**
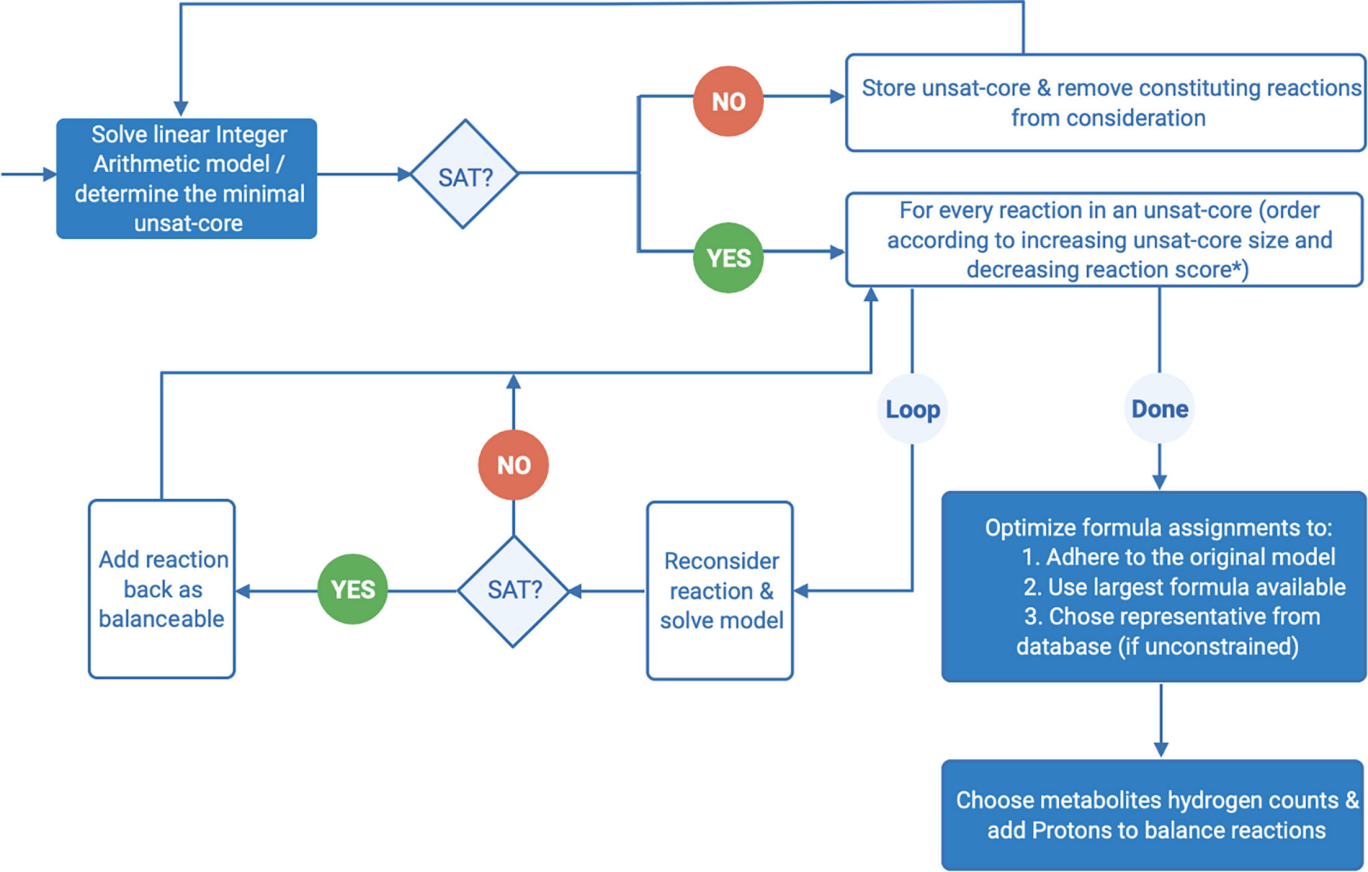
The flowchart of SMT encoding applied in the algorithm.

Figure 1 summarizes the steps above, and the SMT workflow is shown in Fig. 2. These steps led to creating a generic mechanism that integrates various resources—such as databases, servers, software applications, and different services—to simultaneously correct chemical formulas, assign proper charges, and balance reactions. This mechanism is packaged into a Python module called MCC, which is freely available as the Python package and can be accessed from GitHub (see Data Availability).

### Reconstruction of *C. tuberculostearicum*

#### Draft model

The first draft GEM of *C. tuberculostearicum* strain DSM 44922 was reconstructed using CarveMe (36), including the fbc package, and the available genome sequence from NCBI (45) with RefSeq (24) assembly accession, GCF_013408445.1. This draft served as an SBML Level 3 Version 1 (63) template. The input genome file was aligned to the universal FASTA file using DIAMOND (46). The resulting GEM of *C. tuberculostearicum* includes 1,481 reactions and 1,019 metabolites. It is encoded by 622 genes, of which 73 are essential. The GEM identifier, *i*CTUB2024RM, follows recent community naming recommendations (64), where *i* represents *in silico*, “CTUB” is the KEGG (28) abbreviation for the modeled organism, “2024” marks the year of creation as an iteration identifier, and “RM” indicates the lead author of this manuscript. The MeMoTe score of *i*CTUB2024RM is 38%.

#### Model curation

To curate *i*CTUB2024RM, we started by establishing GPR associations using genome annotation data, ensuring they were accurately and directly included in the model. GPR maps gene-encoding enzymes to the reactions they catalyze within Constraints-Based Reconstruction and Analysis (COBRA) models (65). During the automated model creation with CarveMe, we used fbc to add chemical formulas for all metabolites. When multiple chemical formulas were available, we prioritized compounds with higher carbon content and without R group(s). All metabolite charges were manually assigned using the libSBML (40) fbc package. Initially, we performed a web request from the BiGG universal model (66) to assign these charges.

##### Metabolite, Reaction, and Gene annotation

The initial annotation of model components within the draft GEM primarily relied on information from the BiGG Models Database (26). To enhance this, we added references from additional sources, including KEGG (28), MetaNetX (27), ModelSEED (41), BioCyc (22), ChEBI (29), and KBase (67). Reactions and metabolites were annotated using ModelPolisher (68), assigning CV-Terms with the biological qualifier BQB_IS. These terms linked each model component to its corresponding entry in the referenced databases. We utilized the GeneBank (69) file of *C. tuberculostearicum* for gene annotation, extracting both old and new locus tags. We then used these tags to query KEGG (28), UniProt (70), and NCBI, mapping the gene identifiers to the genes in our model.

##### Model extension

Our GEM currently includes 789 metabolites involved in 954 enzymatic reactions and 63 transport reactions, all encoded by 405 genes. We compared the model to the closely related *C. tuberculostearicum* strain SK141 using data from BioCyc (22) to refine the model. We created a SmartTable reflecting the genetic content of the target strain via BioCyc (22) features to identify overlaps with the genomic content of our strain. Of the 1,017 enzymatic and transporter reactions found in BioCyc (22) for this strain, 889 were gene-encoded. We used DIAMOND for a homology search, aligning protein sequences from our strain against translated DNA sequences in the BioCyc genome database. This revealed 548 genes with a threshold of at least 95% identity and 577 genes with 80% identity in our model. We then mapped these gene pairs to BioCyc (22) reactions, focusing on those with at least 95% identity, and compared the reactions between the two strains. For each resulting gene, we checked every encoded reaction. We also reviewed all other associated genes with lower sensitivity in such reactions by involving logical expressions in their GPR. We then analyzed chemical formulas for all participating species. This resulted in a match for 192 reactions with similar metabolites, 155 of which were initially identified to be in the model through BioCyc (22) annotation. The gene comparison revealed 205 reactions with identical genes, 140 of which were already available in the model based on BioCyc annotation. These comparisons identified 442 reactions in BioCyc (22) with full gene evidence, 221 of which were new to our model. Therefore, the decisions are which genes possess which function, which reactions should be included, and finally, in which direction these reactions occur. As a result, we added 221 reactions and 285 metabolites to the model and modified the directionality of 355 reactions (including the added ones) based on the BioCyc (22) data. Although these additions increased the number of orphan and dead-end metabolites, we retained them in the model to preserve its integrity as a knowledge-based model. Since MeMoTe does not account for these metabolites in its scoring, their presence does not affect the calculated score.

##### Energy-generating cycles

Such cycles contradict the first law of thermodynamics because they produce energy-carrying metabolites, such as ATP, without nutrient consumption. To create physically reliable models, these EGCs must be eliminated. This is typically done by either constraining the directionality of reactions or removing the reaction from the network, often manually. In our model of strain DSM 44922, we evaluated the production of 15 energy metabolites while preventing any nutrient uptake. When adding a reaction formed an EGC, we first tried constraining its directionality and, if necessary, removed it. However, constraining the directionality can be problematic as it may force reversible reactions to become irreversible, which is a known disadvantage (71). To resolve all EGCs in the extended model *i*CTUB2024RM, we ultimately had to make the reaction GLYCL irreversible.

##### Blocked reactions

We made further improvements to identify blocked reactions in the *i*CTUB2024RM. Blocked reactions display a steady-state flux other than zero under a given medium. This can be determined using FVA to find fluxes that carry no flux. Blocked reactions could be valuable for future model strain DSM 44922 refinements, so we chose to preserve them in the model.

##### Mass and charge balancing

**Fig 3:**
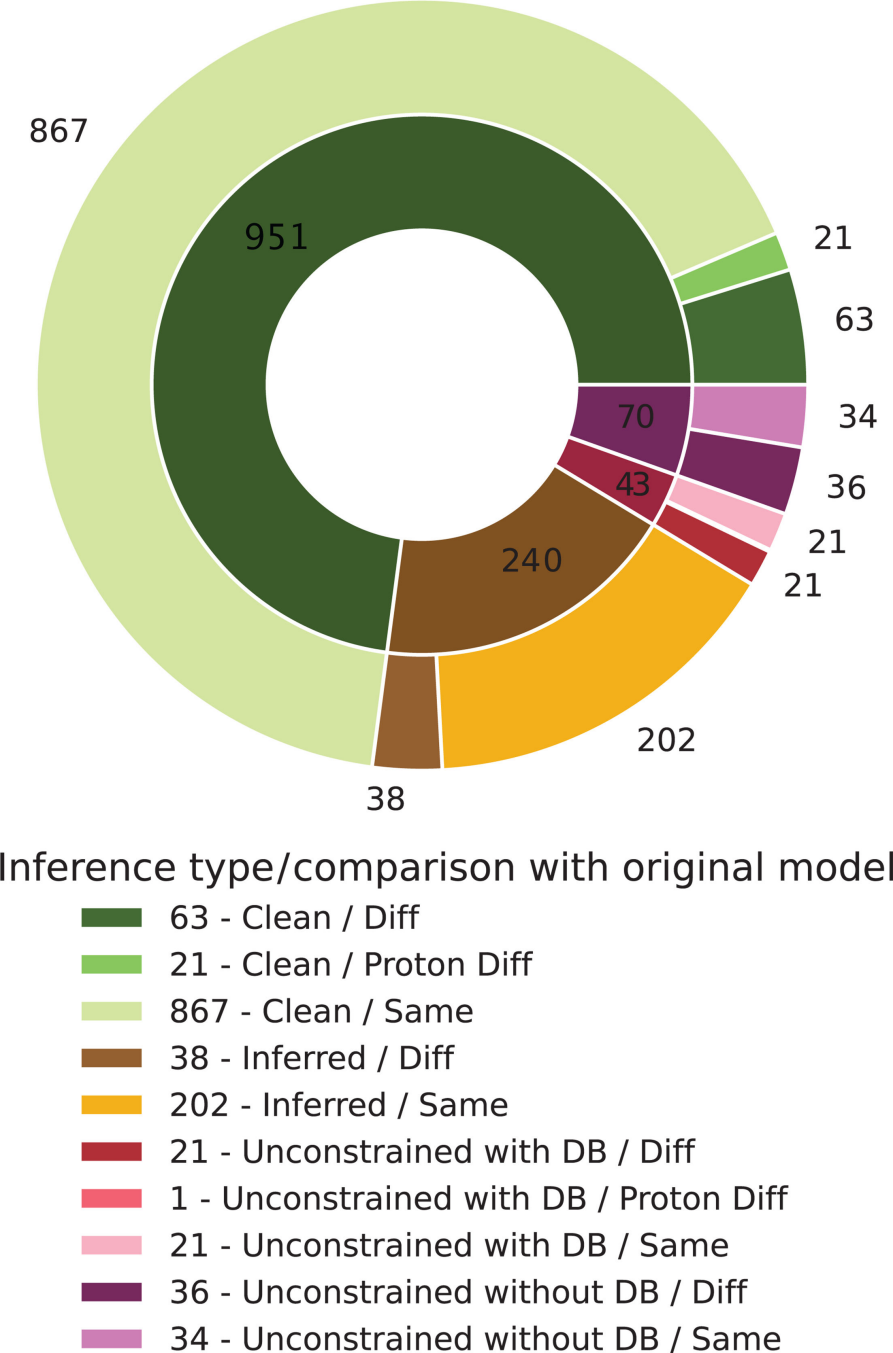
Visualization of MCC applied to the model *C. tuberculostearicum* strain DSM 44922. Metabolite formulas were compared with original formulas from 42 out of 109 initially imbalanced reactions, computed in less than 1,200 s. The pie chart categorizes the assigned metabolite formulas: Green indicates formulas from a database without undefined side-group symbols (Clean), yellow represents metabolites with formulas inferred from clean formulas and reactions (Inferred), and red/purple represents formulas still containing undefined side-group symbols (Unconstrained). The outer ring highlights deviations from initially assigned formulas, where applicable.

To identify the best-matching chemical formulas associated with charges, correct charges associated with metabolites, and achieve mass balance in reactions whose metabolites take part in more than one simultaneous process, all reactions and metabolites’ annotations were required to be added to the SBML (33) file of *C. tuberculostearicum* using the CV terms. Then, our Python module MCC was applied to simultaneously correct the chemical formulas, assign appropriate charges, and balance mass in reactions by comparing data across multiple databases. This process generated a visual report (see Fig. 3) detailing the number of imbalanced reactions and the total running time.

The module’s output was summarized into reaction and metabolite reports (File S1 and S2). The relevant descriptive keywords used in these reports can be found in Tables 1 and 2. The reaction report provides detailed information about imbalanced reactions, with each table column (File S2) offering insights to help correct the model and serve as a starting point for manual curation.

**TABLE 1:** The relevant descriptive table of the reaction report that the module MCC produces

Table title	Description
Imbalanced type	This table column denotes how the reaction is problematic. This shows whether the reaction is mass- or charge-imbalanced or whether many protons are added to the reaction in the balancing process.
Reason	For imbalanced reactions, this gives all reactions that caused this specific reaction to be excluded from our balancing efforts. The reactions listed here could not be balanced. Thus, this reaction was chosen to be taken out. In contrast, for reactions with many protons added, this denotes how many protons were added.
Shared metabolites	This column represents the IDs of metabolites that all reactions in the “Reason” column share. This might likely be an indication of the problem.
Mass difference	Difference in the mass of reactants and products.
Charge difference	Difference in the charge of reactants and products.

**TABLE 2:** The relevant descriptive table of the metabolite report that the module MCC produces

Table title	Description
Inference types	Clean: Formulae that came from a database and had no wildcard symbol. Inferred: Formula for which no clean formula could be found but could be inferred from clean formulas via reactions. Unconstrained: Formula for which no clean formula could be found.
Determined formulas/charges	This shows which formula and charge were selected.
Previous formulas/charges	This shows whether the determined formula and charge are similar to one already assigned in the model.
Used databases	This indicates the determined formula was driven from which database.
Previous databases	In contrast, this shows which database supports the previous formula assigned in the model.
Similarity	Same: The determined formula and charge are the same as the original formula and charge. Proton Diff: The term is used when the change is the addition or removal of protons (H) while affecting the charge correction with the same net difference in protons. Diff: The term is used when the change involves the addition or removal of protons (H), but the proton difference does not account for the change in the charge.

Like the reaction report, we can also take a more detailed look at the formulae assigned to the metabolites through the metabolite report (File S2). Additionally, we are allowed to pass a different model to compare our assignments in this step.

As shown in the reaction and metabolite report tables (File S1 and S2), the module effectively facilitated mass and charge balancing while identifying the reactions that could not be balanced and the reasons. It also highlighted metabolites that caused mass and charge balancing issues due to their chemical formulas or charges. The reports indicate which parts of MCC require manual attention. Consequently, starting manual curation after using the module requires significantly less effort than working directly with the draft model. In particular, formulas without “Used databases” in the metabolite table are likely the most promising targets for further manual refinement. This table column helps users make additional improvements to enhance the model.

##### Redundancy in the model

First, the model was further refined by removing redundancies such as repeated reactions, metabolites, and genes. Additionally, the compartments were streamlined, retaining only three: cytosol, periplasm, and extracellular space. All identifiers in the notes field were moved to the annotation section using the biological qualifier type BQB_IS.

##### SBO and ECO terms

SBO terms were added to the model to categorize genes, metabolites, and reactions using a controlled vocabulary. Each element was assigned a specific SBO term.

ECO terms were also included during the biocuration process to describe evidence and assertion methods, categorizing them based on the functionality of gene products, such as available G-protein-coupled receptors, spontaneous, or none.

##### KEGG pathways and grouping

CV terms with the biological qualifier type BQB_OCCURS_IN, in addition to those declaring identity, were used to indicate the occurrence of a reaction within a specific pathway. These CV terms were implemented using KEGG (28) annotations to extract the relevant pathways.

Furthermore, the SBML Level 3 (37) group’s extension (34) was used to add groups for each KEGG (28) pathway in our model, with annotations linked to the corresponding pathways via identifiers.org (https://identifiers.org/) (42). This allowed us to group reactions by the pathways they participate in, enhancing the model’s reproducibility and reusability for others.

### Growth examination

Reconstructed models may contain inconsistencies due to knowledge gaps about metabolites and reactions, which can hinder their ability to accurately predict growth under different environmental conditions. The model *C. tuberculostearicum* strain DSM 44922, reconstructed using CarveMe and refined further, successfully represented a default growth rate of 3.25 mmol/(gDW⋅h) by setting appropriate constraints (a lower bound of 10 mmol/(gDW⋅h) and an upper bound of 1,000 mmol/(gDW⋅h)) for all exchange reactions.

#### Growth on SNM3

Since *C. tuberculostearicum* strain DSM 44922 has been observed in the human nose (5, 72) it should grow on Systems Network Model 3 (SNM3). To simulate this growth, the exchange bounds were set to −10 and 1,000 mmol/(gDW⋅ h) except for oxygen: −20 and 1,000 mmol/(gDW⋅h) and iron: −0.1 and 1,000 mmol/(gDW · h). However, the model *i*CTUB2024RM could not produce any growth on SNM3 under these settings, so we searched for the minimal supplementation required to support growth. Adding -nicotinamide mononucleotide (NMN) and cytidine-monophosphate (CMP) to the medium enabled growth of 0.85 mmol/(gDw⋅h). Whether NMN is available in human nasal fluid (73) remains unclear. By querying databases, we identified nicotinate NAC as a potential substitute for NMN in the human nose, which can be taken up via diffusion. We, therefore, added the corresponding reactions (as shown in Table 3) to our model.

**TABLE 3:** Corresponding reactions of nicotinate

BiGG ID	Descriptive name	Reaction
NACtpp	Nicotinic acid transport via diffusion	nac_p → nac_c
NACtex	Nicotinic acid transport via diffusion (extracellular to periplasm)	nac_e → nac_p
EX_nac_e	Nicotinate exchange	nac_e ⇌ ∅

Breadth-first search (BFS) is an algorithm that explores nodes in a tree or graph level by level, starting from a root node. It examines all neighboring nodes at the current depth before moving to the next level, making it ideal for finding the shortest path in unweighted graphs (74). Using this, we identified a pathway in the BiGG database that connects to one of the exchange reactions in the medium, allowing *i*CTUB2024RM to produce CMP in SNM3. We restricted our search to BiGG reactions with gene evidence related to CMP. Although we found several reactions, none could support the optimal growth objective for *C. tuberculostearicum* or cause futile cycles in the model. Therefore, the only solution was to add the exchange reaction EX_cmp_e to the medium.

In summary, *C. tuberculostearicum* can be grown in SNM3 either by adding NMN and CMP to the medium or by incorporating the reactions listed in Table 3 in addition to the following reaction into the model and adding NAC and CMP to the medium.

MQL8M (MQL8 Maintenance Reaction):


mql8_c⟶mqn8_c+h_c

#### Growth on LB and M9

The growth of *i*CTUB2024RM was tested in LB medium. The model did not predict growth in this medium unless supplemented with NMN. With NMN supplementation, *i*CTUB2024RM achieved a growth rate of 1.08 mmol/(gDW⋅h). In M9 medium, growth was also supported at 0.7 mmol/(gDW⋅h) when NMN, cmp, and β-alanine (ala_B) were added.

#### Minimal growth medium

We computed the minimal set of metabolic reactions required to sustain the minimum optimal growth rate for *C. tuberculostearicum*, as detailed in Table 4. The growth rate achieved with this minimal medium was 0.26 mmol/(gDW⋅h). It is important to note that this minimal medium is not unique as it is determined through optimization. To find the minimal number of additional open exchange reactions that support a biomass objective function of 1/h, we used an optimization approach with a lower bound of 10 mmol/(gDW⋅h) for all exchange reactions (59). These minimal reaction sets will help examine growth rates and validate the model in future experiments.

**TABLE 4:** The minimal components required for the growth of *C. tuberculostearicum* with the relevant descriptive name extracted from the BiGG Models Database (66)

Common exchange reactions	Descriptive name
EX_cmp_e	CMP exchange
EX_cl_e	Chloride exchange
EX_k_e	Potassium exchange
EX_ca2_e	Calcium exchange
EX_mg2_e	Magnesium exchange
EX_mn2_e	Manganese exchange
EX_cobalt2_e	Co^2+^ exchange
EX_zn2_e	Zinc exchange
EX_cu2_e	Cu^2+^ exchange
EX_pnto_R_e	(R)-Pantothenate exchange
EX_fe3_e	Fe^3+^ exchange
EX_cys L_e	L-Cysteine exchange
EX_nmn_e	NMN exchange

#### Growth on different carbon sources

To enhance the potential for experimental validation of our *in silico* predictions, we simulated the growth rate of the *C. tuberculostearicum* model using the minimal medium with different carbon sources. The minimal medium outlined in Table 4 used initially L-cysteine as the carbon source. We then tested the minimal medium by replacing L-cysteine with the carbon sources listed in Table 5. However, our computational analysis revealed that the *C. tuberculostearicum* strain DSM 44922 model could not grow on any carbon sources, as shown in Table 5.

**TABLE 5:** The carbon source compounds replaced by L-cysteine to examine the growth of *C. tuberculostearicum,* with the relevant descriptive name extracted from the BiGG database (66), each causing *C. tuberculostearicum* to grow at 0 mmol/(gDW⋅h)

Common exchange reactions	Descriptive name
EX_ac_e	Acetic acid
EX_cit_e	Citric acid
EX_glyc3p_e	D,L-α-Glycerol phosphate
EX_ala_D_e	D-Alanine
EX_fru_e	D-Fructose
EX_fum_e	Fumaric acid
EX_arg_L_e	L-Arginine
EX_asn_L_e	L-Asparagine
EX_asp_L_e	L-Aspartic acid
EX_glu_L_e	L-Glutamic acid
EX_gln_L_e	L-Glutamine
EX_his_L_e	L-Histidine
EX_glyc_e	Glycerol
EX_gly_e	Glycine
EX_orn_e	L-Ornithine
EX_phe_L_e	L-Phenylalanine
EX_pro_L_e	L-Proline
EX_ser_L_e	L-Serine
EX_ala_L_e	L-Alanine
EX_glc_D_e	α-D-Glucose
EX_ptrc_e	Putrescine
EX_pyr_e	Pyruvic acid
EX_succ_e	Succinic acid
EX_4abut_e	γ-Amino butyric acid
EX_ile_L_e	L-Isoleucine
EX_mal_L_e	L-Malic acid
EX_lac_L_e	L-Lactic acid
EX_leu_L_e	L-Leucine
EX_mnl_e	D-Mannitol
EX_etha_e	2-Aminoethanol
EX_glcur_e	D-Glucuronic acid
EX_sbt_D_e	D-Sorbitol
EX_xyl_D_e	D-Xylose
EX_gly_asp_L_e	Glycyl-L-aspartic acid
EX_met_L_e	L-Methionine
EX_drib_e	2-Deoxy-D-ribose
EX_val_L_e	L-Valine
EX_malt_e	Maltose
EX_inost_e	L-Histidine
EX_rib_D_e	D-Ribose
EX_thr_L_e	L-Threonine
EX_gly_glu_L_e	Glycyl-L-glutamic acid
EX_gly_pro_L_e	Glycyl-L-proline
EX_but_e	Butyric acid
EX_lys_L_e	L-Lysine
EX_ppa_e	Propionic acid
EX_man_e	D-Mannose
EX_tre_e	m-Tartaric acid

#### Functional pathway recovery: tuberculostearic acid (TBSA) biosynthesis

**Fig 4:**
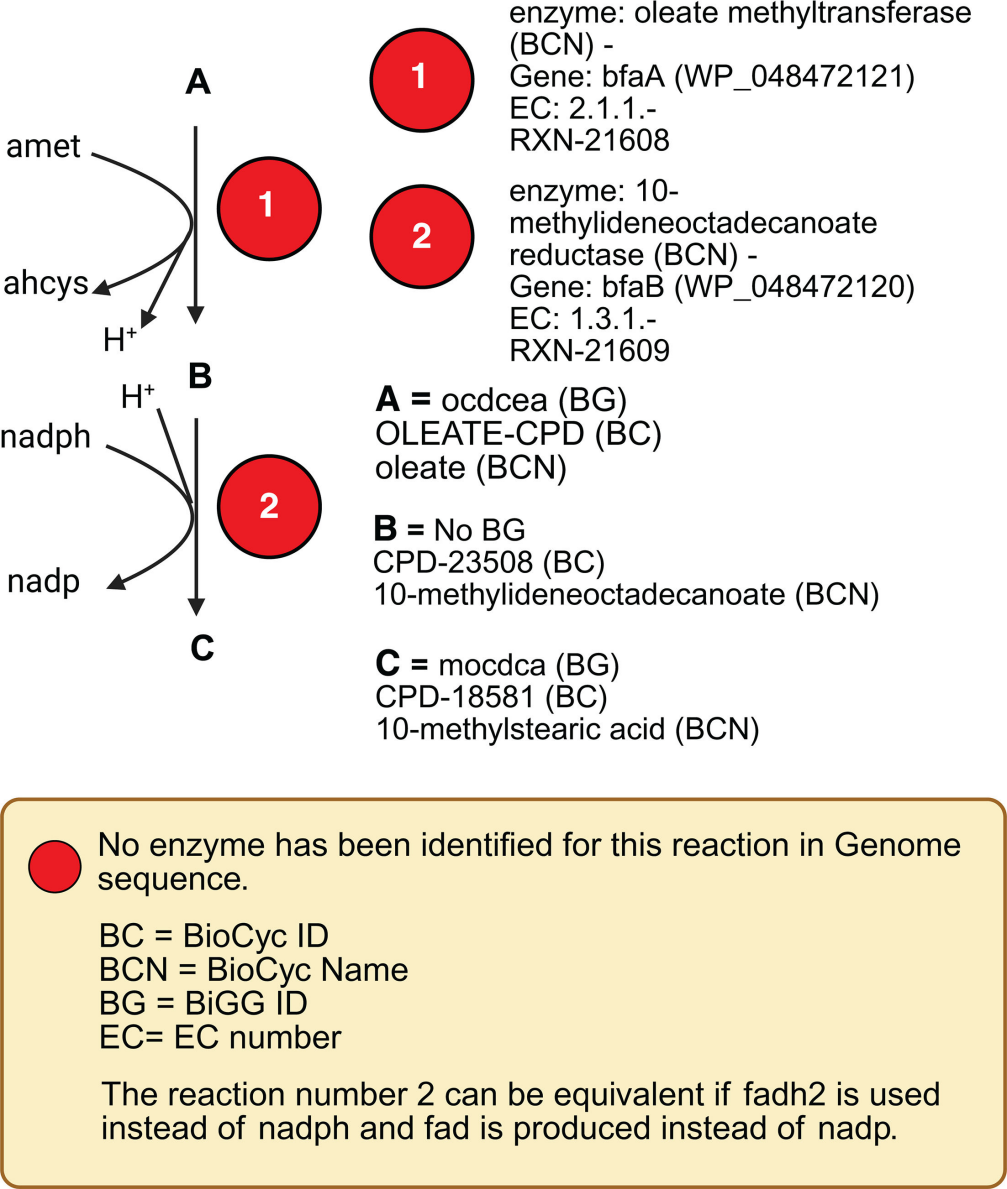
Two reactions involved in the 10-methylstearic biosynthesis pathway based on the BioCyc database.

The biosynthesis of tuberculostearic acid (TBSA), identified by 10-methylstearic acid in *Corynebacterium tuberculostearicum*, is not fully characterized. However, based on studies in related bacteria, the production of TBSA involves a two-step enzymatic process: SAM-dependent methylation of the double bond and reduction of the methylene intermediate to TBSA (75). These reactions are shown by BioCyc IDs RXN-21608 and RXN-21609, respectively, and are only available in BioCyc (22), but not in the BiGG database (26). These reactions are encoded by bfaA and bfaB, as shown in Fig. 4.

There has been some indirect evidence that two such enzymes, encoded by the umaA and ufaA1 genes of *Mycobacterium tuberculosis*, can catalyze the conversion of oleate to 10-methylstearic acid when expressed in *E. coli* (76, 77). However, the reduction that must occur during this process has not been explained, and the results could not be reproduced (78). None of the available models for *C. tuberculostearicum* indicated any of those reactions and genes, including AGORA, BioCyc, or KEGG. Our model also did not include these reactions due to the absence of relevant genes to catalyze them. However, the KEGG database lists a gene in *C. tuberculostearicum*, named I6I74_06555, encoding a long-chain fatty acid—CoA ligase. This enzyme activates long-chain fatty acids by converting them into acyl-CoA derivatives, a step that could be involved in fatty acid metabolism, including TBSA biosynthesis. Our model includes this reaction, which is encoded by the same gene indicated in the KEGG database.

Due to insufficient reactions involved in the production of TBSA, we apply two approaches to perform a simulation of this production. One approach we use is testing whether the model can synthesize TBSA, adding a demand reaction. We then simulate a biologically meaningful drain by maximizing the yield of net production of TBSA. Next, we check pathway closure, doing gap-filling to temporarily test if this metabolite can be routed into or out of the model by introducing a sink reaction. This simulation analysis uses BHI broth or agar (ATCC Medium 44) supplemented with 1% Tween‑80. Multiple studies have confirmed that TBSA production of *C. tuberculostearicum* under these conditions requires lipid supplementation for robust growth.

This supplementation is necessary to activate the fatty acid–CoA ligase and subsequent methylation/reduction steps. In contrast, SNM3 comprises amino acids, vitamins, salts, and glucose but does not include lipids or fatty acids, especially oleic acid. Existing literature indicates that TBSA production relies on lipid supplementation, which SNM3 lacks. Therefore, there is no evidence that *C. tuberculostearicum* produces TBSA when grown in SNM3. While none of the studies have explicitly measured TBSA concentrations in *C. tuberculostearicum*, except literature from 1984 by cultivation bacteria (7), the findings from *Mycobacterium* and the other genus *Corynebacterium* strongly indicate the necessity of exogenous oleic acid to activate the fatty acid–CoA ligase and drive the SAM-/FAD-dependent steps toward TBSA biosynthesis.

To simulate TBSA production, we first defined an *in silico* version of the BHI broth supplemented with 1% Tween‑80 (7), as detailed in the supplementary table (File S5). However, *C. tuberculostearicum* strain DSM 44922 did not produce any growth in this medium. To enable growth, we identified the minimal set of metabolites required to supplement the medium. Interestingly, BHI produced the same results as SNM3 concerning the missing metabolites for growth. Supplementing the medium with NMN and CMP enabled growth at the rate of 0.48 mmol/(gDW⋅h). Next, we introduced two reactions associated with the TBSA pathway to the model. A demand reaction (DM_mocdca_c) was then introduced to optimize the TBSA production. This approach successfully enabled TBSA synthesis at 4.09 mmol/(gDw⋅h). These simulation results suggest that the genome of the modeled organism lacks two essential genes encoding enzymes required for TBSA production.

Since metabolic fluxes can exist in non-growth conditions, mainly if the network allows thermodynamically feasible pathways, GEMs can produce metabolites via demand, sink, or exchange reactions without biomass production (79). Based on this, we simulated the model in the BHI medium without supplementing the metabolites required for growth. This demonstrates that the BHI model can synthesize TBSA under nutrient-limited conditions without active cell growth. It highlights the presence of feasible flux distributions through TBSA biosynthetic pathways that do not necessarily contribute to biomass formation.

**Fig 5:**
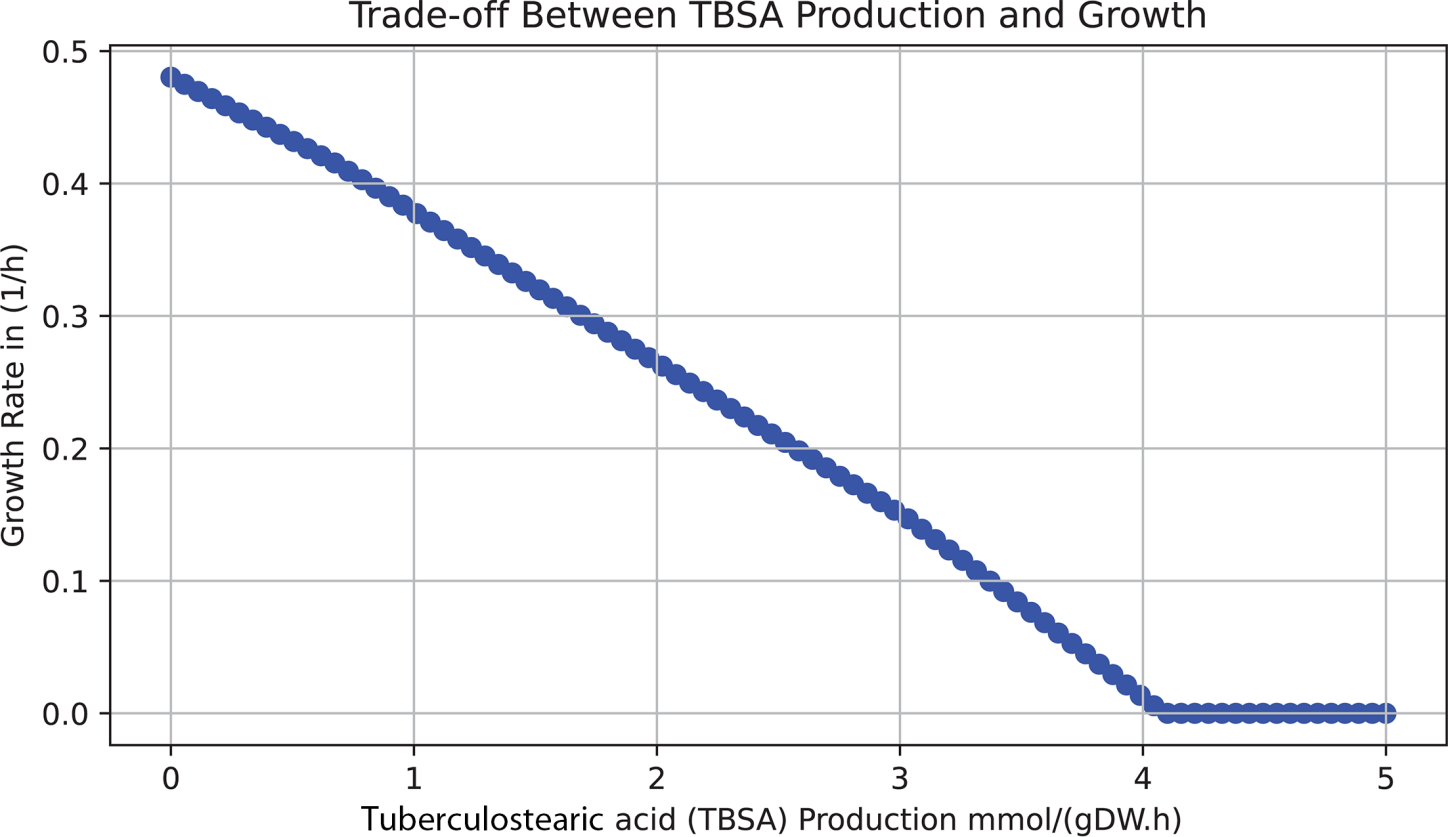
Trade-off between TBSA production and growth in brain-heart infusion (BHI). The demand reaction for TBSA was set as the objective function to investigate the relation between the production of TBSA and growth. The growth rate varied between a maximum growth rate of 0.480 mmol/(gDw⋅h) and 0 mmol/(gDw⋅h) while TBSA production was between 0 mmol/(gDw⋅h) and 4.09 mmol/(gDw⋅h).

Next, to analyze the trade-off between growth and TBSA production, as shown in Fig. 5, we reactivated the model’s growth in BHI by supplementing the required metabolites. The plot indicates a negative correlation. When TBSA production is low, growth is high; as TBSA production increases, the growth steadily declines.

**Fig 6:**
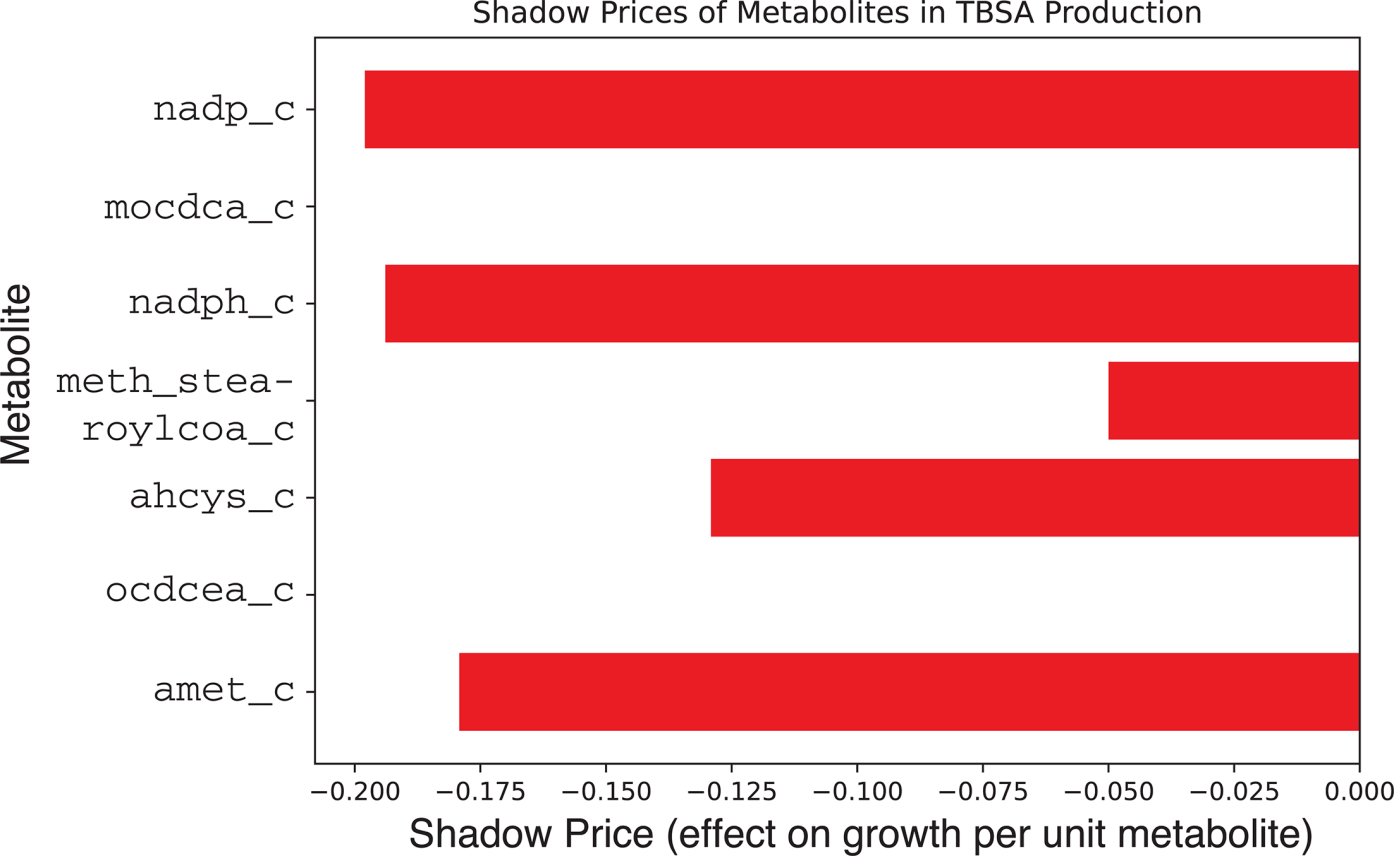
The shadow price of internal metabolites involved in TBSA maximization. These metabolites are limiting which biomass affects the objective function.

Since TBSA is a product not included in the growth reaction, we used shadow pricing to assess how changes in precursor availability affect the objective function. As shown in Fig. 6, several internal metabolites are limiting for TBSA maximization. Given that the growth rate of *C. tuberculostearicum* in BHI is about ten times lower than the maximum possible TBSA production rate, a balanced objective function, defined as a weighted linear combination of growth and TBSA production, can be used. This balanced objective yields reduced TBSA production.

### Python module validation

**Fig 7:**
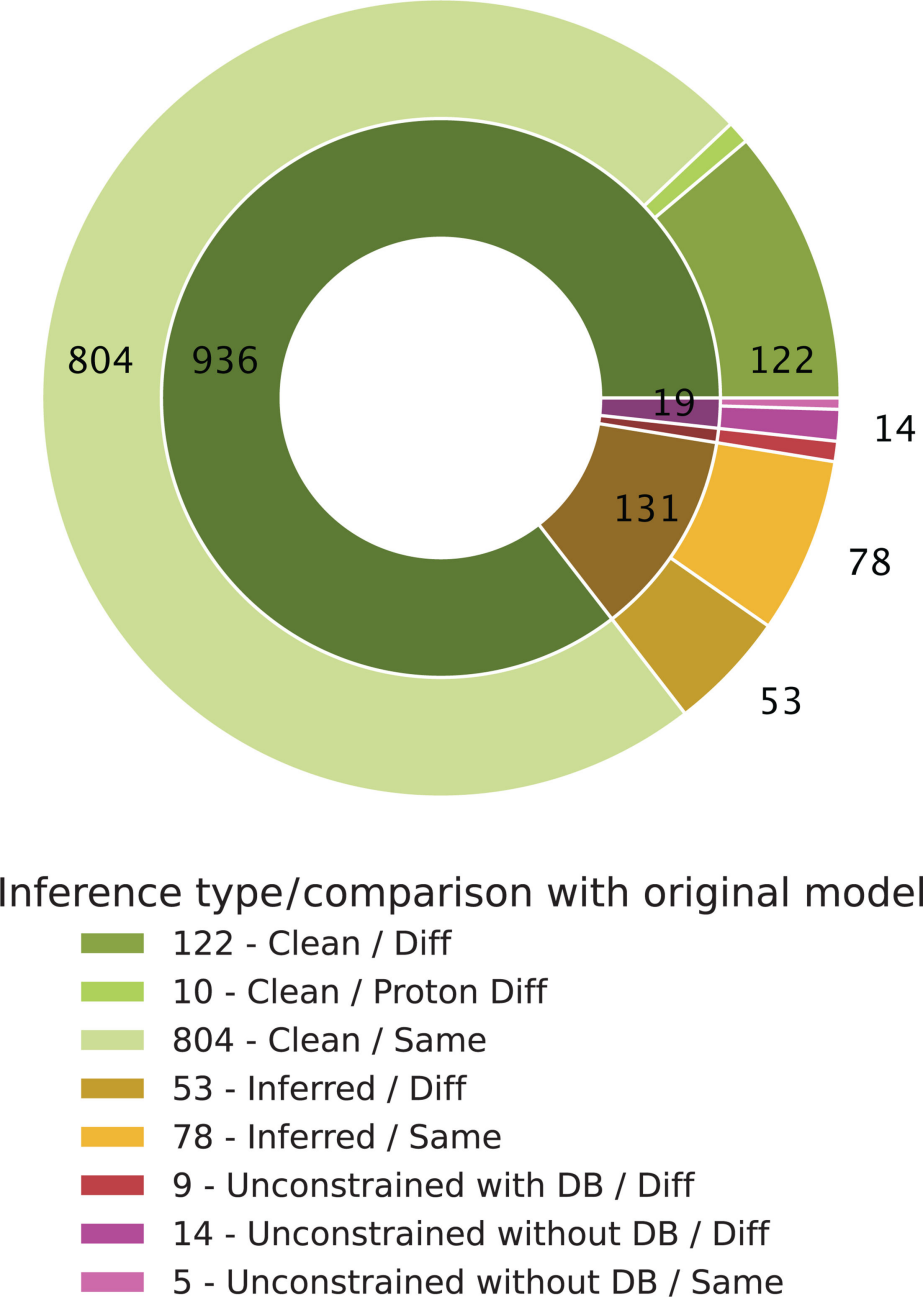
Validation of MCC applied to the model *Dolosigranulum pigrum* strain 83VPs-KB580 before manual curation for mass and charge balancing. The pie chart illustrates the assigned metabolite formulae in the early model compared to the manually curated version. Green represents formulae curated by MCC, showing 804 chemical formulae that match the target model exactly. In contrast, 122 are different while selected from a database without undefined side-group symbols. Yellow indicates formulae that contain an undefined side-group symbol inferred from clean formulas and reactions, highlighting both similarities and differences. Red/purple denotes formulae that still contain undefined side-group symbols where a definitive formula could not be determined.

To validate the Python module, we applied the algorithm to the GEMs of *Dolosigranulum pigrum* strain 83VPs-KB5 (80). This model was reconstructed using the genomic sequence from NCBI (81) via the accession code ASM19771v1. We used an early version of the GEM for *D. pigrum* before mass and charge balancing had been manually curated. Next, we compared the MCC-curated model of *D. pigrum* to the published model manually curated by Renz et al. Despite manual efforts, the published model still contains 321 imbalanced reactions, including 182 pseudo-reactions, leaving 139 imbalanced reactions. As shown in Fig. 7, our module significantly reduced the number of imbalanced reactions, though some issues remained. Details of the MCC-curated model and the corresponding draft model of *D. pigrum*, not a manually curated version of the same organism, are provided in the supplementary tables (File S3 and S4).

These metabolite assignments were sensible in context, with most differences concentrating on metabolites that are otherwise difficult to assign manually. These include cases where metabolite annotations are ambiguous or complex, often resulting in nonsensical or overlooked assignments during manual curation. Therefore, this variation occurred because our module adheres closely to the original model and databases, leading to different decisions about balancing reactions. Additionally, the module can systematically evaluate and integrate information from various databases, while manual curation is prone to human error and limited by the curator’s ability to cross-check multiple sources simultaneously. This ensures a more comprehensive and consistent reconstruction process. These choices can vary when manually adjusting the module using the option for fixed reactions and metabolite parameters. Thus, the module not only provides more consistent results but also addresses gaps that may arise from human oversight. A comparison between the MCC-curated model of *D. pigrum* and the manually curated one is provided in the supplementary table (File S6). For instance, only 57 chemical formulas, 36 charges, and 54 both in metabolites were assigned that differed from the target model curated manually. The differences in chemical formulas were sometimes reported due to the number of differences in H, i.e., C_6_H_8_N_3_O_4_P against C_6_H_9_N_3_O_4_P. After using MCC, starting manual curation could offer significant advantages.

To further compare an MCC-curated model with existing gold-standard GEMs (20), we define two models: A and B. Model A represents the version before manual mass and charge balancing, while Model B reflects the version after manual curation. We then apply MCC to Model A to produce Model C. By comparing Models B and C, we can gain a comprehensive understanding of how MCC performs relative to manual curation of the mass and charge balance. This approach enables a fair and meaningful comparison, which is shown in the fact that using this strategy, we could only apply MCC to the *D. pigrum* model (80) as we had access to the model at each refinement stage. In contrast, only the final published versions of the models are available to us (20). To demonstrate the refinement capabilities of MCC despite the above-mentioned limitation, we applied MCC to five generated models (20), three models from AGORA (21) and two models from CarveMe (36), to observe the improvements it produces. Models from AGORA (21) were *Bacillus subtilis subsp. subtilis str. 168*, *Corynebacterium tuberculostearicum SK141*, and *Escherichia coli str. K-12 substr. MG1655*. *Corynebacterium pseudotuberculosis C231* and *Mycobacterium tuberculosis H37Rv* were chosen from CarveMe (36). All models needed further refinement steps to reach a high-quality GEM, which is outside the scope of this publication. We only applied MCC to each model while removing extra strings on metabolite and reaction IDs to see how its MeMoTe score improved. This means that only the mass and charge balance was taken into account. This was a challenge as the annotations in all models were not complete. The MeMoTe score for annotation in AGORA (21) models was less than 50%, and for CarveMe (36) models, it was less than 25%. This caused several issues. In the AGORA (21) models, many metabolites lack annotation, and we could not find them in databases via searching. Examples include i17tcaacgam_c, ai17tcaacgam_c, and others with a high negative charge and long chemical formulas. Since these metabolites were not found in any databases and no further information was available regarding their origin, MCC could not determine their chemical formulas and, using an inferred approach, assigned them a charge of 0. Despite those differences, the model’s MeMoTe score after using MCC in AGORA (21) models stayed the same. However, the MCC results on models created by CarveMe (36) were notable as these models did not have any correct charges; all assigned charges from CarveMe were zero. Therefore, annotations play an important role in correcting mass and charge balancing when applying MCC to existing models as MCC relies on databases. This is particularly important when the models were extended using multiple resources and experiment-based literature.

### Biological relevance and functional insights from the MCC-curated model

Reconstructing and analyzing GEMs is a powerful systems biology approach. Its applications range from a basic understanding of genotype-phenotype mapping to solving biomedical and environmental problems. However, the biological insights gained from these models are limited by multiple heterogeneous sources of uncertainty, which are often challenging to quantify (82). Mass and charge balancing represents a fundamental aspect of constructing GEMs and is a key source of uncertainty. They are important in extracting meaningful biological insights, especially for pathogenic species or complex ecosystems. Applying mass balancing approaches to a metabolic network results in a convex mathematical representation of linear equations and inequalities that define an underdetermined model (83). It ensures the model reflects actual biochemistry, making it reliable for simulation, prediction, and intervention design. An imbalance in mass and charge conflicts with conservation laws, thermodynamic feasibility, and accurate predictions. All reactions in the model are balanced in atomic mass and charges, enhancing its robustness in predicting metabolic fluxes concerning proton balancing. Imbalanced reactions can generate fluxes without energy or metabolite input, which did not occur in our model due to the refinement step, which corrected energy metabolism. Without proper charge balancing, models can falsely predict metabolic adaptations to nutrient availability and misrepresent key biochemical strategies. For instance, even minor stoichiometric imbalances in biomass and the model’s structure can lead to significant predictive errors because the exact stoichiometry of each biochemical reaction imposes a mass conservation constraint that must be maintained in a steady state (84, 85). An unbalanced model can misidentify candidate enzymes for antimicrobial therapy, produce misleading biomarkers or virulence factors, and falsely simulate pathogen behavior in host-pathogen interaction. A GEM study on *Setaria viridis* in plants compared a mass and charge-balanced model with an unbalanced variant, which affected a mispredicted utilization uptake source (86). To demonstrate how this balancing enhances the biological insights derived from the model, we analyzed several GEMs before and after applying MCC. The results are reported as a comparative list in Table 6.

**TABLE 6:** A comparison of the growth rate and TBSA production on models in different stages of reconstruction before and after applying MCC^*a*^

Model name	Default growth	Growth on SNM3	Growth on BHI	TBSA production
Model 1	36.48	0.76	0.99	4.28
Model 2	36.48	0.76	0.99	4.28
Model 3			0.70	4.19
Model 4	47.77	0.81	0.90	13.12
Model 5	46.84	1.05	0.62	3.66
Model 6	46.84	0.85	0.48	4.09

^*a*^ Models often predict growth rates, and unbalanced reactions can distort flux distributions and reduce the predictive accuracy. In our model, the growth rates on different media and TBSA production predicted at various stages of reconstruction prior to applying MCC, compared to the posterior of MCC, are sometimes higher or lower. This highlights the strong corrective effect of MCC on improving flux balance and enhancing the accuracy of growth rate predictions. Only annotations were modified from Model 1 to Model 2 and did not affect the computational result. The results changed from Model 2 to Model 3 due to the application of MCC. From Model 3 to Model 4, the results are different due to the extension of the model from Model 4 to Model 5; some refinement and steps have been done, including removing redundancy, which affects the result. From Model 5 to the final Model, MCC was applied again, and the result appeared to vary as expected. Model 1 = CarveMe generated. Model 2 = Metabolite, Reaction, and gene annotation assigned—SBO terms assigned—ECO term assigned. Model 3 = MCC applied. Model 4 = An extension of the model by comparing it to another strain in BioCyc, and hits over 90 % were added. Model 5 = further refinements (including using SBOannotator, adding KEGG pathway, adding Group Plugin, correcting ECO terms, correcting EGC, correcting all annotations, and removing redundancy) added in the extension model. Model 6 = final model (MCC applied again).

### MeMoTe comparison

**Fig 8:**
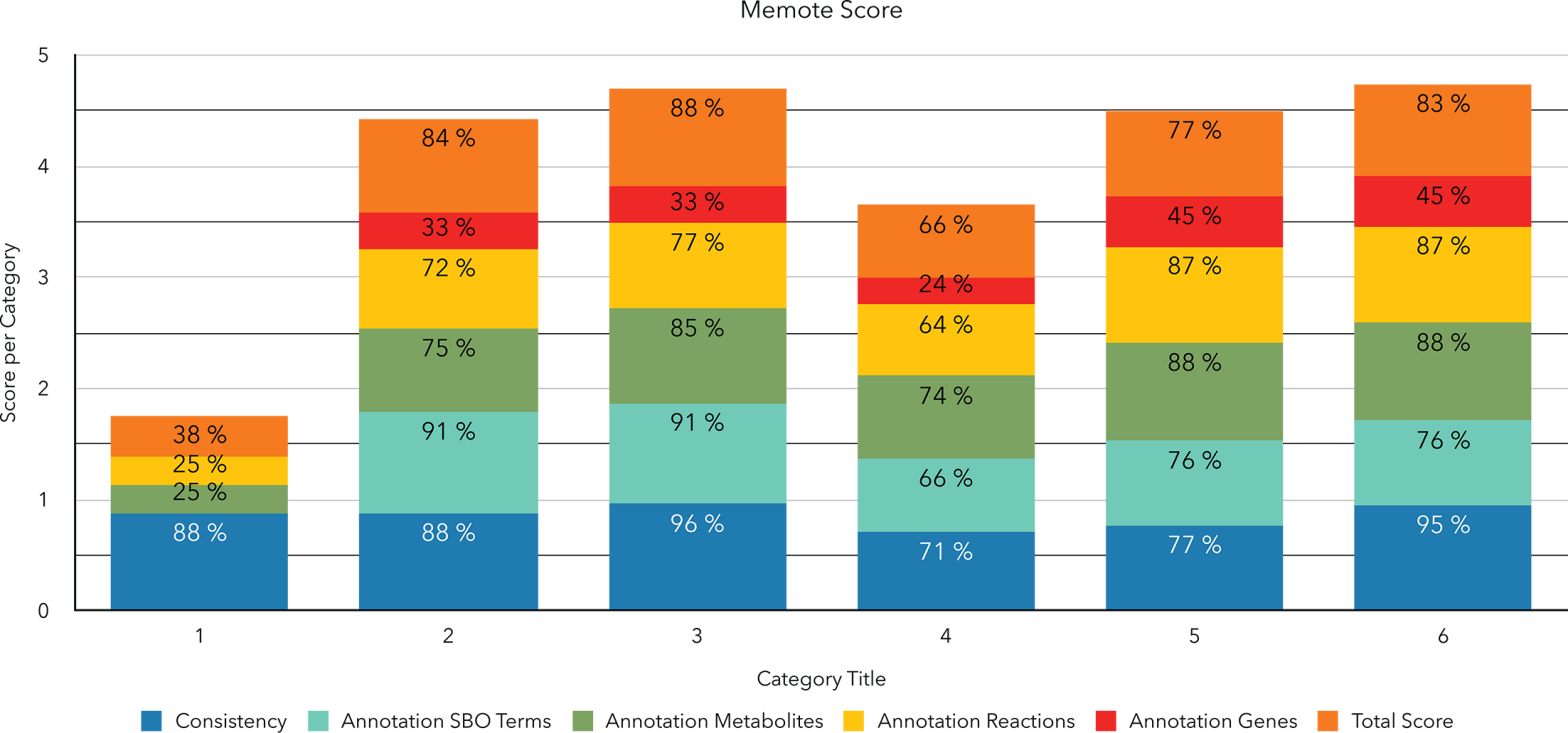
The MeMoTe scores illustrate the model's improvement after curation and refinement. Applying MCC enhanced the model's consistency as MeMoTe is the result of stoichiometric consistency (SC), mass balance (MB), charge balance (CB), metabolite connectivity (MC), and unbounded flux (UF) in the default medium. The score is calculated as follows: . Although extending the model initially reduced the score due to adding reactions, metabolites, and genes, which affected mass and charge balance, further refinements improved the model's quality. MCC helped achieve 95% consistency and a final total score of 83%.

An overview of the MeMoTe score during the curation step is shown in Fig. 8 and Table 7. This indicates additional refinements enhanced the model’s quality, with MCC bringing it to 95% consistency and a final total score of 83%. The supplementary tables (File S1 and S2) result from the MCC-curated model of *C. tuberculostearicum* that highlights areas where manual mass and charge balance can be further optimized. By using the following commands, the user can get the assignments that differ from the original report or all assignments that are not supported by any database:

metabolitereportdf[metabolitereportdf["Similarity"] != "Same"]

metabolitereportdf[metabolitereportdf["Used Databases"] == ""]

**TABLE 7:** Category No 5 includes using SBOannotator, adding KEGG pathway, adding Group Plugin, correcting ECO terms, correcting EGC, correcting all annotations, removing redundancy, and applying MCC

Category	Title	Consistency	Annotation metabolites	Annotation reactions	Annotation genes	Annotation SBO terms	Total score
1	CarveMe generated	88%	25%	25%	0%	0%	38%
2	Metabolite, reaction, and gene annotation assigned; SBO terms assigned; ECO term assigned	88%	75%	72%	33%	91%	84%
3	MCC applied	96%	85%	77%	33%	91%	88%
4	Extension of the model by comparing to another strain in BioCyc and hits over 90% added	71%	74%	64%	24%	66%	66%
5	Further refinements added in the extension model	77%	88%	87%	45%	76%	77%
6	Final model (MCC reapplied)	95%	88%	87%	45%	76%	83%

## DISCUSSION

Recent studies (2, 5, 7, 12, 15, 87) highlight the significance of *Corynebacterium tuberculostearicum* in both medical and industrial fields. It plays a role in the human microbiome and can act as an opportunistic pathogen, making it important for understanding bacterial infections and developing targeted therapies. However, we lack comprehensive information on the nasal microbiome and uncultured microbes *in vivo*. Additionally, members of the *Corynebacterium* genus are well known for industrial applications, particularly in producing amino acids like glutamate and lysine (88). While *C. tuberculostearicum* strain DSM 44922 has not been extensively studied for these purposes, understanding its metabolic mechanisms could reveal its potential and biotechnological value. To explore this, a GEM of *C. tuberculostearicum* was developed as a comprehensive tool for simulating and understanding its metabolic pathways. GEMs can grant the key steps toward understanding the principles of microbes and offer crucial insights into microbial behavior. They open up a fascinating scenario of possible novel discoveries if constructed in a high-quality, manually curated manner, which is time-consuming. The model reconstructed is essential for identifying the organism’s metabolic capabilities, vulnerabilities, and interactions within the microbiome and guiding metabolic engineering in industrial processes, especially toward TBSA production.

We initially constructed the model using CarveMe (36), while adhering to high-quality reconstruction criteria. CarveMe (36) typically ensures growth by adding reactions using a gap-filling approach, which means some reactions may be included that are not directly derived from genome annotations. Further refinement and manual curation were necessary to achieve accurate modeling. Recent studies have developed many automated network reconstruction tools to accelerate the reconstruction process and reduce the probability of human errors (65, 89–93). Manually correcting chemical formulas, charge annotations, and balancing reaction masses is one of the most complex and time-intensive steps in the reconstruction process. This complexity makes it essential to automate the process efficiently, especially when annotation details for metabolites and reactions are included in the model. Several software platforms have been developed to streamline this step in the reconstruction process. For instance, the SuBliMinaL toolbox (94), written in Java, uses third-party tools to retrieve necessary data from databases such as KEGG (28) and MetaCyc (44) automatically. It balances charge and mass by considering different stoichiometric coefficients and uses mixed-integer linear programming (MILP) (94). Similarly, MetRxn relies on existing GEMs as references for charge calculation, using linear programming (LP) to minimize discrepancies between reactant and product charges, as well as compatibility with MATLAB (95). Other approaches, like the “MIP” procedure introduced by Chan et al. (96), employ optimization techniques to resolve elemental balance inconsistencies. Despite these advancements, mass and charge balancing remains a significant bottleneck in the network reconstruction process.

To overcome and streamline this process, we developed a Python module called MCC that efficiently gathers relevant data from various databases and simultaneously corrects chemical formulae, charges, and mass-balancing reactions. MCC collects mass and charge data from multiple databases such as BiGG (26), MetaNetX (27), KEGG (28), BioCyc (22), and ChEBI (29) and analyzes the distribution of metabolites based on different formula-charge combinations and the support each combination receives from these databases. Our module offers a significant advantage by consolidating information from multiple sources and consistently applying it, particularly for chemical formulas. It aims to reproducibly find the most accurate and consistent assignments of formulas and charges, even when dealing with conflicting resources, while remaining as close to the existing model as possible. Additionally, the module provides easily interpretable visualization and logging tools for comparing changes, allowing users to review and verify modifications rather than just accepting them. This feature also highlights areas where the module may have struggled, offering insights for further manual curation. Users uncertain about a formula can trace its origin and see which metabolites are affected. Unlike existing tools, which may overlook conflicting chemical formulas or refer to manual curation, our module identifies imbalanced reactions, unclear formulas, and the level of support each assignment has from various databases. An exciting feature of the module is its ability to accept a default definition by allowing users to provide a dictionary of fixed assignments for specific metabolites. For example, if a user specifies the chemical formula of water as H2O with a charge of 1, the module will maintain this setting during the refinement process. Unlike tools such as SuBliMinaL (94), which balances reactions by considering different stoichiometric coefficients, our module focuses on consistency and adds protons only as needed to achieve balance. Thus, SuBliMinaL would be more complementary than redundant with the module. However, a limitation is that our algorithm relies solely on the balance within the model and cannot distinguish between correct and incorrect formulas if poor sources are used. With good information, though, the module enforces consistency, which enhances accuracy. Technically, the only check for accuracy is the balance, without an additional plausiblity check. Our module’s validation strategy demonstrated approximately 92% accuracy in matching information with a well-curated model, specifically by evaluating the number of clean formulae. The validation process was straightforward to understand.

**Fig 9:**
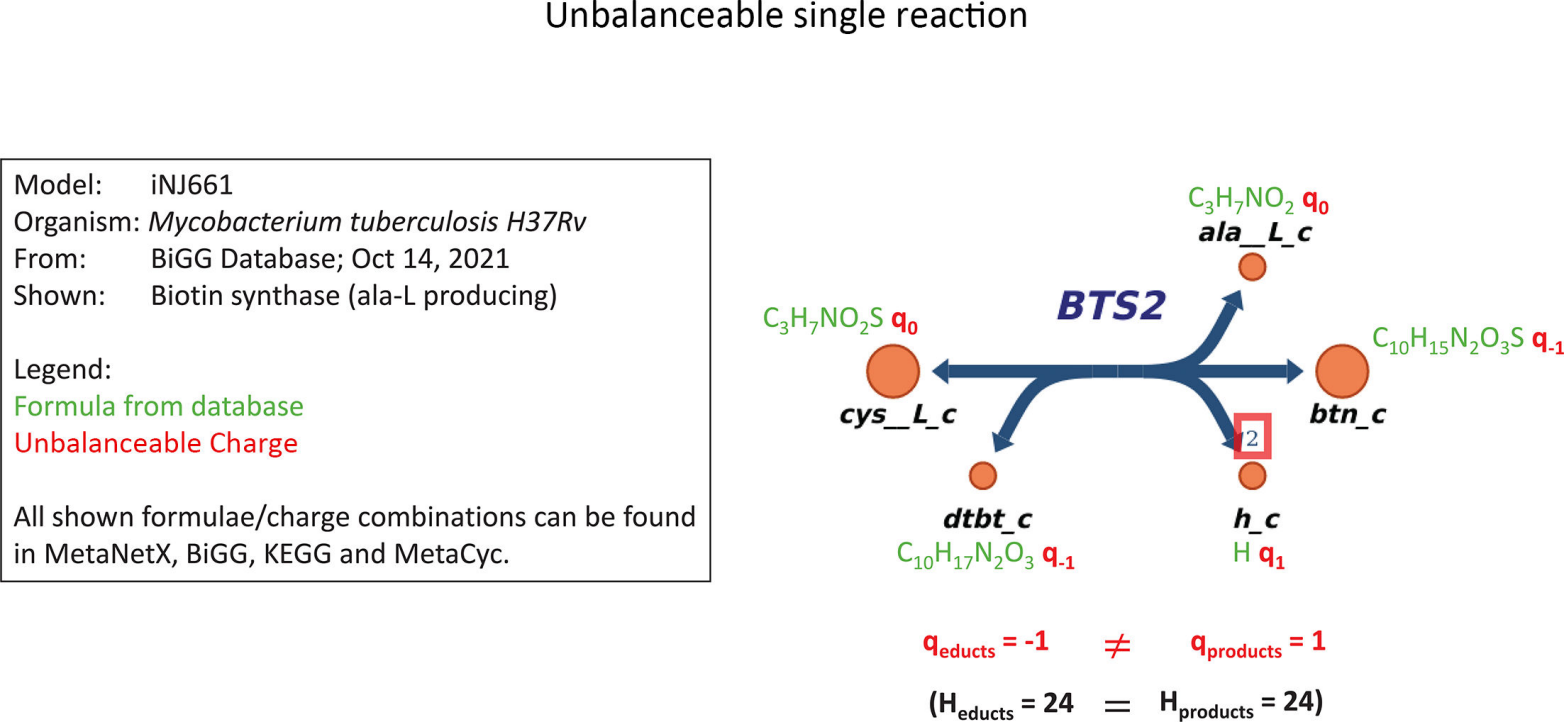
An example of an unbalanced single reaction due to incorrect charge assignments from databases.

**Fig 10:**
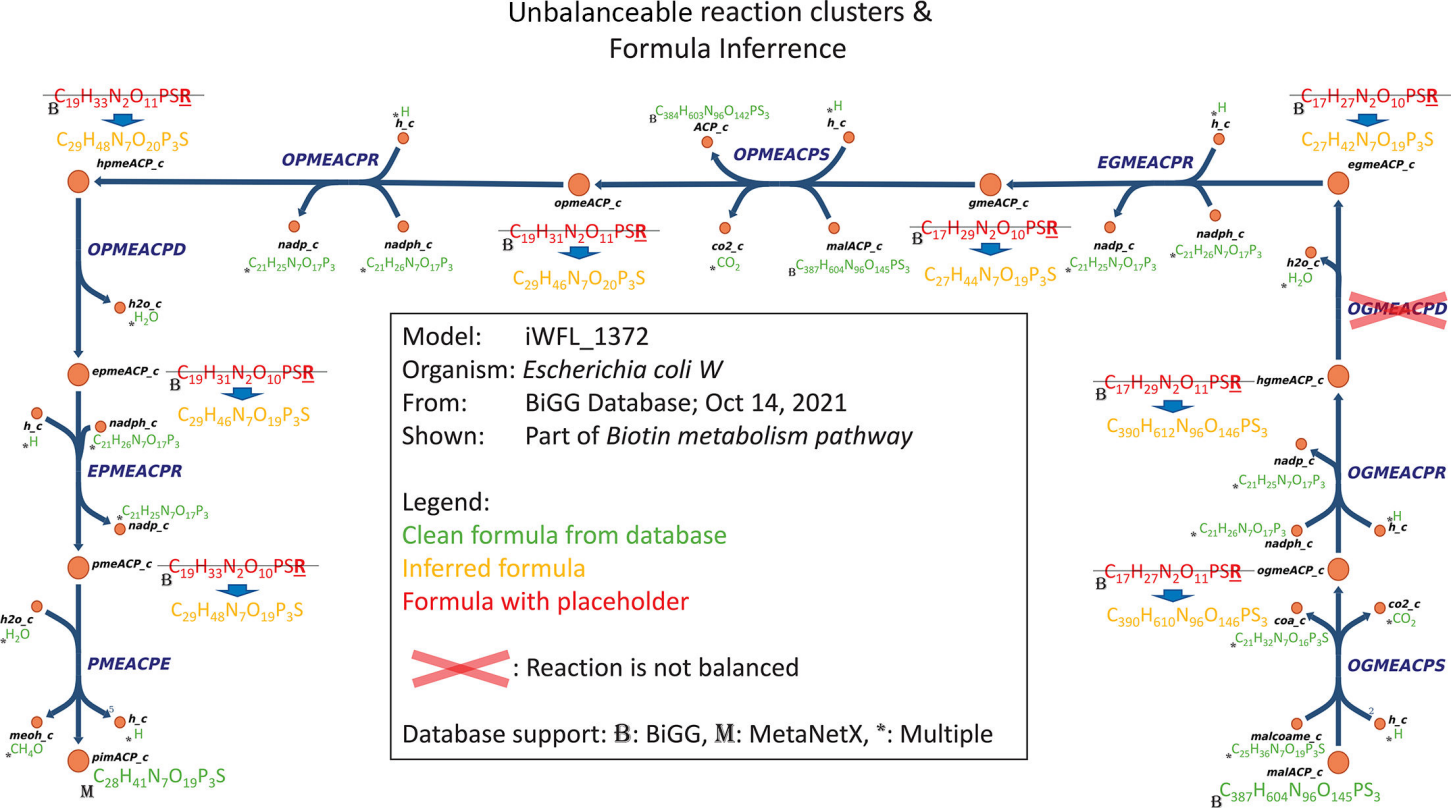
An example of an unbalanced reaction clusters and incorrect formula inference due to erroneous chemical formulae assigned by databases.

We tested the MCC tool on a model before precise manual curation of mass and charge balancing. The comparison between using MCC and manual curation showed that while manual curators could achieve accurate results, they often had to stop once they reached the best possible outcome. This was likely due to the difficulty in consistently finding matching information for the remaining mass and charge balancing. Given the time-intensive nature of manual curation, our findings suggest that MCC could serve as an effective alternative, offering greater efficiency without compromising the accuracy. For those seeking a fully balanced model, manual curation can be continued after using MCC, using its suggestions for further refinement. However, achieving 100% balancing remains challenging due to ongoing database conflicts and unresolved symbols in formulae. Despite this, MCC provides a strong foundation for the process. Despite the advantages of using MCC, we identified metabolites involved in interconnected reactions with inconsistencies in their chemical formulas and charges. When attempting mass balancing from one side of these reactions, we encountered incompatibilities in assigning correct charges and formulas to the participating metabolites when approached from the other side. Figures 9 and 10 illustrate examples of conflicting charges and formulas, respectively. Improvements include resolving rest groups by creating new metabolites representing each possible version.

Analyzing the nasal microbial community is a complex process with significant implications as the species inhabiting this environment remain understudied or difficult to culture using standard laboratory media (97–99). For instance, in 1984, the bacterium *C. tuberculostearicum* was first identified in cases of diphtheria (7). Later, it was linked to CRS (12), showing a higher abundance in CRS patients compared to controls (5). Recently, it has also been associated with skin cells (15). Despite its importance in human health, the mechanisms by which *C. tuberculostearicum* influences these conditions are poorly understood. To explore the role and function of *C. tuberculostearicum* in the upper respiratory tract (URT), we first need to construct an *in silico* model of this nasal inhabitant. By using constraint-based modeling toolboxes, we can advance their application. An integrated approach in the reconstruction process could produce a high-quality model that includes elements from microbiology, ecology, and evolution. Based on its significance in the nasal microbial community, we have constructed a high-quality GEM of *C. tuberculostearicum* strain DSM 44922 to frame investigations into specific biological questions. To facilitate the reconstruction process, we developed a Python module called MCC to streamline the mass and charge balancing step. This user-friendly module can be applied to any GEM. MCC is crucial because it includes core benefits as follows: (i) It automates a typically manual, error-prone, complex, and time-consuming task even for experienced modelers. (ii) By integration of biochemical data from multiple trusted sources, it allows for evidence-based resolution of reaction inconsistencies and increases the annotation coverage. (iii) It uses standardized evaluation metrics for model quality (e.g., number of balanced reactions and annotation density), which help users assess improvements over draft models and compare them across organisms or pipelines. (iv) It supports less-curated or novel organisms, where tools like ModelPolisher (68) or manually curated templates are not available or applicable, and (v) last but not least, MCC outputs structured logs and reports, making all curation steps traceable and auditable for transparency and reproducibility, thereby improving the accuracy, reliability, and usability of GEMs for both research and industrial purposes. This high-quality GEM allows us to systematically simulate metabolic potential and generate testable hypotheses regarding growth conditions, which can direct future experimental design where conventional tools are lacking (97, 100). These hypothesis-driven discoveries were shown when we analyzed the growth rate of *C. tuberculostearicum* strain DSM 44922 in default, SNM3, and BHI media plus TBSA production on models before and after applying MCC.

Our Python module will aid in reconstructing high-quality GEMs as more experimental data and consistent databases become available. As database conflicts are resolved, there is potential for further improving the MCC module. Future technologies will likely enhance these tools, enabling the construction of increasingly accurate models for target organisms. We hope that future studies continue to test the hypotheses generated by the *C. tuberculostearicum* strain DSM 44922 model on the growth achievement and provide feedback for refining the model, thus closing the loop between computational models and experimental validation and significantly reducing computational costs. Furthermore, alternative mechanisms might produce TBSA through this process since our model includes fatty acid metabolism. It is also possible that this strain-specific process does not produce TBSA at all. However, no experimentally validated alternative approach has been validated as of now.

## Data Availability

The genome-scale metabolic model of C. tuberculostearicum strain DSM 44922 is freely available from BioModels Database (101) under accession number MODEL2407310001 as a COMBINE archive file (102) comprising a model in SBML 1206 Level 3 Version 1 (65) format with extension package fbc version 2 (40) and annotation (103). The Mass and Charge Curation software is freely available under the Massachusetts Institute of Technology (MIT) license terms from https://github.com/draeger-lab/MassChargeCuration/ or via the Python Package Index (PIP). MCC is freely available via PIP, from https://github.com/draeger-lab/MassChargeCuration/. The model iCTUB2024RM can be obtained as an SBML file wrapped in an OMEX archive from BioModels Database, accession MODEL2407310001.

## SUPPLEMENTAL MATERIAL

Supplemental material S1 spectrum.03200-24-s0001.csv Reaction report *Corynebacterium tuberculostearicum* model.

Supplemental material S2 spectrum.03200-24-s0002.csv Metabolite report *Corynebacterium tuberculostearicum* model.

Supplemental material S3 spectrum.03200-24-s0003.csv Reaction report *Dolosigranulum pigrum* model.

Supplemental material S4 spectrum.03200-24-s0004.csv Metabolite report *Dolosigranulum pigrum* model.

Supplemental material S5 spectrum.03200-24-s0005.csv Brain heart infusion with Tween 80.

Supplemental material S6 spectrum.03200-24-s0006.csv Formula charge mismatches *Dolosigranulum pigrum* manual mass charge balance and MCC.

ASM does not own the copyrights to Supplemental Material that may be linked to, or accessed through, an article. The authors have granted ASM a non-exclusive, world-wide license to publish the Supplemental Material files. Please contact the corresponding author directly for reuse.
